# HER3-targeted protein chimera forms endosomolytic capsomeres and self-assembles into stealth nucleocapsids for systemic tumor homing of RNA interference *in vivo*

**DOI:** 10.1093/nar/gkz900

**Published:** 2019-10-16

**Authors:** Felix Alonso-Valenteen, Sayuri Pacheco, Dustin Srinivas, Altan Rentsendorj, David Chu, Jay Lubow, Jessica Sims, Tianxin Miao, Simoun Mikhael, Jae Youn Hwang, Ravinder Abrol, Lali K Medina Kauwe

**Affiliations:** 1 Department of Biomedical Sciences, Cedars-Sinai Medical Center, Los Angeles, CA 90048, USA; 2 Department of Chemistry and Biochemistry, California State University, Northridge, CA 91330, USA; 3 Department of Information and Communication Engineering, Daegu Gyeongbuk Institute of Science and Technology, Daegu, Korea; 4 Geffen School of Medicine, University of California, Los Angeles, CA 90095, USA

## Abstract

RNA interference represents a potent intervention for cancer treatment but requires a robust delivery agent for transporting gene-modulating molecules, such as small interfering RNAs (siRNAs). Although numerous molecular approaches for siRNA delivery are adequate *in vitro*, delivery to therapeutic targets *in vivo* is limited by payload integrity, cell targeting, efficient cell uptake, and membrane penetration. We constructed nonviral biomaterials to transport small nucleic acids to cell targets, including tumor cells, on the basis of the self-assembling and cell-penetrating activities of the adenovirus capsid penton base. Our recombinant penton base chimera contains polypeptide domains designed for noncovalent assembly with anionic molecules and tumor homing. Here, structural modeling, molecular dynamics simulations, and functional assays suggest that it forms pentameric units resembling viral capsomeres that assemble into larger capsid-like structures when combined with siRNA cargo. Pentamerization forms a barrel lined with charged residues mediating pH-responsive dissociation and exposing masked domains, providing insight on the endosomolytic mechanism. The therapeutic impact was examined on tumors expressing high levels of HER3/ErbB3 that are resistant to clinical inhibitors. Our findings suggest that our construct may utilize ligand mimicry to avoid host attack and target the siRNA to HER3^+^ tumors by forming multivalent capsid-like structures.

## INTRODUCTION

HER3/ErbB3 promotes the growth of an expanding range of tumor types (1–13). An increase in its expression is associated with a worsening prognosis and a more aggressive phenotype that resists current clinical interventions, including inhibitors of the ErbB receptor kinase axis (1,2,7,14–18). Accordingly, there is growing interest for the targeting of HER3 in the clinic. Although it contains an inactive kinase domain, making it an impractical target for signal inhibition (19,20), the increased density of HER3 on the surfaces of resistant tumor cells provides a useful biomarker for active targeting of those cells and a potentially valuable portal for the accumulation of ErbB-directed therapeutic-loaded nanocarriers. In support, we have shown that our HER3-homing protein construct, HPK, mediates the targeted delivery of chemotherapeutic compounds to trastuzumab (Herceptin)-resistant breast tumors, which display high cell surface densities of HER3 (21). As these tumors can resist conventional tumoricidal drugs, the targeted delivery of alternative cargo—such as small interfering RNA (siRNA)—may provide useful therapeutic options. The delivery of siRNA via HER3-mediated targeting has not yet been reported, likely because HER3 is a recently emerging tumor target and corresponding ligands are not widely available or widely explored for directing therapeutic carriers.

RNA interference (RNAi) offers a powerful gene-silencing tool for cancer treatment, but *in vivo* delivery barriers limit its clinical application (22). In addition to targeting the RNAi to tumor cells, effective *in vivo* delivery vehicles must package the RNAi molecules such that they are protected from nuclease-mediated degradation during transport and also penetrate the target cells to release the cargo into the cytoplasm (23,24). Cytoplasmic delivery is essential for selective pairing with and degradation of mRNA targets (22). Hence, a robust system is needed for *in vivo* targeted delivery of RNAi. Although lipid-enveloped and bioconjugated siRNAs have been extensively explored in preclinical studies (25), many of the existing technologies lack selective tumor targeting and do not target HER3. Thus, in the present study, we tested the utility of HPK for directing RNAi to HER3-dense tumors *in vivo*.

The potent delivery of nucleic acids by viruses has inspired the development of virus-derived proteins (26)—including HPK—for the nonviral transport of therapeutics. The proteins comprising the outer shell, or capsid, of many viruses mediate cell penetration during infection (27). Viral capsid-derived proteins retain cell-penetrating activities of whole viruses without infection activity or the potential for recombination with wild-type viruses (28). Their use for *in vivo* non-viral delivery of nucleic acids may require covalent attachment of chemical moieties to reduce immune surveillance and protect the cargo from systemic degradative molecules (26). However, such chemical modifications may alter the function and complicate vector construction, creating challenges for translation to the clinic (29). We created viral capsid-derived biocarriers using a single chimeric fusion construct, HPK, containing the functions for cargo loading, tumor targeting, and membrane penetration (21). HPK is derived from the adenovirus (Ad) capsid penton base (PB) protein, which contributes to membrane penetration and cell entry of the virus during infection (30–32). The capsid PB has been explored for the delivery of molecular therapeutics because of its ability to penetrate the cellular endosomal membrane (32–36) despite its unclear mechanism for endosomal disruption, or endosomolysis. The ligand used to target HPK to tumors is derived from the minimal receptor-binding region of the ErbB growth factor, heregulin-1α1 (37). This minimal ligand specifically recognizes HER3 and induces rapid endocytosis while reducing heregulin-mediated signaling in HER3-expressing tumor cells (21). These findings extend to HER2^+^ breast tumor cells (38,39) due to the coexpression and heterodimerization of HER3 and HER2 (20,40–42). These studies demonstrated that HPK mediates robust uptake of drug compounds into cells (21) and thus may bear the features needed for effective transport of siRNA payloads.

To test HPK as a HER3-targeted biocarrier for systemic delivery of siRNA to tumors *in vivo*, we focused on two major areas of translational development. First, we used complementary approaches to uncover several key mechanisms that are crucial for *in vivo* delivery, including the vaguely understood mode of endosomolysis by the PB that is essential for the delivery of nucleic acids (31,33). Here, we used computational biophysical methods for the structural modeling of HPK in different oligomeric states and then evolved the HPK structures under physiological conditions using molecular dynamics (MD) simulations to uncover its influences on siRNA particle assembly, stability, targeting, and host recognition, which were then tested in functional assays. Second, our assessment of targeted siRNA delivery by HPK entailed a validation of appropriate mouse models for interpreting targeting *in vivo*. As most clinically approved targeted therapies are specific to human antigens, caution must be applied when evaluating tumor targeting in rodent models with human xenografts, which is often disregarded when nanocarriers are tested for ligand-directed targeting. Here, we examined the interspecies cross-reactivity of HPK to determine the extent to which *in vivo* targeting can be validly interpreted. We also assessed HPK function in two different *in vivo* models of HER3-expressing cancer, including an immune-competent model of triple-negative breast cancer. HER3^+^ tumors present clinical scenarios with limited therapeutic recourse (43); hence, the delivery of siRNA therapeutics via HER3-mediated targeting may widen the options available to such cancers.

## MATERIALS AND METHODS

### Materials

A synthetic ErbB2/HER2 siRNA duplex (RTF primer, TCTGGACGTGCCAGTGTGAA; RTR primer, TGCTCCCTGAGGACACATCA) was obtained from Invitrogen (Carlsbad, CA, USA). Firefly luciferase (Luc) and negative-control (scrambled [Scr]) siRNAs were obtained from Applied Biosystems/Ambion. Buffer A comprised Dulbecco's modified Eagle's medium, 20 mM HEPES (pH 7.4), 2 mM MgCl_2_ and 3% bovine serum albumin (BSA; in phosphate-buffered saline [PBS] or H_2_O).

### Cells

All cell lines were obtained from the American Type Culture Collection and were maintained at 37°C with 5% CO_2_ under mycoplasma-free conditions in complete medium (Dulbecco's modified Eagle's medium supplemented with 10% fetal bovine serum, 100 U/ml penicillin, and 100 μg/ml streptomycin).

### Protein production

Recombinant proteins were produced from the pRSET-A bacterial expression vector, which adds an N-terminal 6× His sequence for metal chelate affinity purification of the fusion protein. To produce the fusion protein in bacteria, 50-ml cultures of *Escherichia coli* BLR(DE3)pLysS transformed with the pRSET constructs were grown to turbidity at 37°C with vigorous agitation and then expanded to 500-ml cultures that were induced with 0.4 mM IPTG (isopropyl-β-d-thiogalactopyranoside) at an optical density at 600 nm of 0.6–0.8. Three hours later, the cells were pelleted and resuspended in 5 ml lysis buffer (50 mM NaH_2_PO_4_, 50 mM NaCl, pH 8) containing 0.1% Triton X-100 and 1 mM phenylmethylsulfonyl fluoride. After one freeze-thaw cycle, 10 mM MgCl_2_ and 0.01 mg/ml DNase I were added, and the lysates were gently agitated at room temperature (RT) until the viscosity was reduced. The lysates were then transferred to ice, and 1 M NaCl and 10 mM imidazole (final concentrations) were added. The lysates were clarified by centrifugation at 4°C, followed by affinity purification using nickel-charged medium, washing and eluting with a step gradient of imidazole (ranging from 20 to 250 mM) in 50 mM NaH_2_PO_4_ and 300 mM NaCl. Fractions containing eluted protein (∼92 kDa) were buffer exchanged by ultrafiltration in storage buffer (20 mM HEPES [pH 7.4], 150 mM NaCl, 10% glycerol).

### Particle formation

The incubation of HPK with siRNA species (4:1 molar ratio) at RT for at least 20 min and then for at least 1 h on ice with agitation produced complexes we referred to as HSi. The mixtures were subjected to ultrafiltration using 100K molecular-weight-cutoff membranes to isolate assembled particles from unassembled components. The siRNA concentration in particles was quantified by either ethidium bromide staining after gel electrophoresis in comparison to that of free siRNA of known quantities or by heparin treatment to release siRNA followed by fluorescence quantification using a nucleic acid binding dye (RiboGreen; Thermo Fisher Scientific). The protein content was measured by spectrophotometric absorbance using either the Bradford method (Bio-Rad dye assay) or measuring the absorbance at 280 nm.

### Structural modeling

#### Generation of the pentameric HPK structure

For HPK (also known as HerPBK10) (44), the Her and PBK domain structures were based on the following amino acid sequences:

Her, ELLPPRLKEMKSQESAAGSKLVLRCETSSEYSSLRFKWFKNGNELNRKNKPQNIKIQKKPGKSELRINKASLADSGEYMCKVISKLGNDSASANITIVESNEIITGMPASTEGAYVSSESPIRISVSTEGANTSSSTSTSTTGTSHLVKCAEKEKTFCVNGGECFMVKDLSNPSRYLCKCQPGFTGARCTENVPMKVQNQEKAEELYGGSGGSGS (215 amino acids);

PBK, MRRAAMYEEGPPPSYESVVSAAPVAAALGSPFDAPLDPPFVPPRYLRPTGGRNSIRYSELAPLFDTTRVYLVDNKSTDVASLNYQNDHSNFLTTVIQNNDYSPGEASTQTINLDDRSHWGGDLKTILHTNMPNVNEFMFTNKFKARVMVSRLPTKDNQVELKYEWVEFTLPEGNYSETMTIDLMNNAIVEHYLKVGRQNGVLESDIGVKFDTRNFRLGFDPVTGLVMPGVYTNEAFHPDIILLPGCGVDFTHSRLSNLLGIRKRQPFQEGFRITYDDLEGGNIPALLDVDAYQASLKDDTEQGGGGAGGSNSSGSGAEENSNAAAAAMQPVEDMNDHAIRGDTFATRAEEKRAEAEAAAEAAAPAAQPEVEKPQKKPVIKPLTEDSKKRSYNLISNDSTFTQYRSWYLAYNYGDPQTGIRSWTLLCTPDVTCGSEQVYWSLPDMMQDPVTFRSTRQISNFPVVGAELLPVHSKSFYNDQAVYSQLIRQFTSLTHVFNRFPENQILARPPAPTITTVSENVPALTDHGTLPLRNSIGGVQRVTITDARRRTCPYVYKALGIVSPRVLSSRTFKKKKKKKKKK (581 amino acids).

The Her domain structure was obtained by using the protein structure prediction server I-TASSER (45), and the PBK domain structure was built with the SWISS-MODEL (46) server utilizing the PB structure (PDB 3IZO) (47) as a template. The structural models did not include the N-terminal 6 × His tag used for affinity purification. The pentameric PBK structure was built from the monomer structure, obtained through SWISS-MODEL, using the PDB 1X9T (48) as a template. This pentameric PBK structure was placed in a water box and relaxed for 100 ns using MD simulations as described below for the full HPK structure. Pentamer snapshots were saved at regular intervals, and the snapshot with the smallest root mean squared deviation of Cα atoms between structures was selected as an average structure. The Her domain structure from I-TASSER was physically placed near the N-terminal end of the selected average structure of PBK, avoiding any clashes with the PB, which resulted in an HPK pentamer structure. This structure was relaxed in its native environment as described below.

#### Relaxation of pentameric HPK in a physiological environment

The above-described structure of HPK was embedded in a water box (using the tleap program that is part of the AMBER simulation suite (49)) made up of 293 402 water molecules with the system size of 247 Å × 244 Å by 165 Å and a total of 941 411 atoms including the protein and added Cl^−^ ions that neutralize the system. The simulated system is shown in Supplementary Movie S2. The pentameric protein structure was relaxed to test its stability using the following computational protocol, which utilized the pmemd.cuda (GPU) program from the AMBER simulation suite and the AMBER force field ff14SB to represent the interatomic interactions and forces. Step 1, the solvent energy is minimized while keeping protein constrained to enable the solvent to adjust around the protein; step 2, the solvent is relaxed for 100 ps using MD simulation at 310 K temperature and 1 atm, while keeping the protein restrained to enable the solvent to rearrange especially around the polar exposed residues in the protein and to fill any artificial holes in the simulation box; step 3, the full system's energy is minimized to enable the protein to adjust to the solvent; step 4, the full system is heated to 310 K and equilibrated for 100 ps to enable the full system to adjust to the physiological temperature; step 5, the full system is equilibrated for 250 ps to enable the density of the full system to converge; step 6, the full system is equilibrated for 100 ns, during which time the system snapshots are saved at regular intervals for subsequent analysis of the stability and other features of the simulated system. The root mean squared deviation of the Cα atoms of one structure relative to another structure, measured from the snapshots to determine the representative structure, was used to analyze the buried solvent accessible surface area and the relative size of the pentamer versus monomer structures. It was also used in the PropKa server (50) to compute the p*K*_a_ values of all titratable residues in the HPK pentamer.

### Co-precipitation assay

PBK and HPK fusion proteins (10 μg each) containing 6× His tags were prebound to nickel-nitrilotriacetic acid resin (10 μl 50% slurry) in 200 μl incubation buffer (50 mM NaH_2_PO_4_ [pH 8], 0.1 M NaCl, 5 mM imidazole, 10% glycerol, 0.01% NP-40) for 1 h at 4°C. Unbound protein was removed by washing the resin once in incubation buffer and twice in wash buffer (incubation buffer containing 0.05 M NaCl). [35S]Met-labeled PB produced by *in vitro* translation in accordance with the manufacturer's protocol (Promega TNT coupled transcription/translation kit) was added to each mix to achieve a 200-μl final volume in wash buffer and incubated with agitation for 2 h at 4°C. The resin was pelleted and washed four times in wash buffer, and the pelleted resin was resuspended in a mixture of 8 μl water and 8 μl SDS-PAGE loading dye. Mixtures were boiled for 5 min, followed by pelleting and loading of supernatants onto the gel.

### Receptor binding

Enzyme-linked immunosorbent assay (ELISA) plate wells were coated with HER3 peptide at ∼5 μg/ml (100 μl per well) overnight at 4°C in coating buffer (0.1 M NaHCO_3_, pH 9.6) and then washed in PBS to remove unbound protein. The plates were incubated for 1 h at RT in blocking buffer (3% BSA in PBS). Aspirated wells then received HPK (∼0.5 μg/well) ± preadsorbed HER3 peptide (i.e. peptide preincubated with HPK at 10× molar excess) in PBS and were incubated for 1 h at RT with agitation, followed by washing and processing for immunodetection using an anti-RGS-His tag antibody (1:1000; Qiagen, MD, USA) and anti-mouse secondary antibody (1:2000). To analyze HPK binding after HER3 silencing, 96-well plates were seeded with 5000 MDA-MB-435 cells 24 h before a HER3 siRNA pool (product number 1027416; Qiagen) was applied using standard transfection procedures with commercial formulations. Briefly, 100 ng of HER3 siRNA or scrambled control siRNA was diluted in Opti-MEM medium and delivered using Lipofectamine 3000 (Invitrogen) according to the manufacturer's instructions. Twenty-four hours later, the cells were treated with 1.5 μg of HPK per well diluted in complete medium. HPK was added to cells on ice for 1 h to promote binding but not uptake. Cells were then thoroughly washed with PBS, fixed using 4% PFA, and processed for immunodetection of HPK and HER3 as described under ‘Immunocytofluorescence/Immunohistofluorescence’. High-throughput acquisition of HER3 and HPK signals from each cell (>500 cells per field in three independent fields per well of triplicate wells) was performed using an ImageXpress Pico scanner (Molecular Devices).

### Cell surface ELISA

Cells were plated at 1 × 10^4^/well unless otherwise indicated in 96-well plates and maintained for 24 h before washing with PBS containing 1% MgCl_2_ and 1% CaCl_2_ fixing without permeabilization (to detect cell surface proteins only), and ELISA processing, as described previously (44). The plates were then processed for crystal violet staining for normalization according to cell number, as described previously (51). Cell surface HER3 was detected on human tumor lines using an antibody that recognizes the extracellular domain of HER3 (Ab105; Pierce-Thermo Fisher, MA, USA). Mouse HER3 was detected on 4T1 cells and in specimens using an antibody that cross-reacts with both human and mouse HER3 (1B2E; Cell Signaling Technologies). Other receptor levels (ErbB1/HER1, ErbB2/HER2, and ErbB4/HER4) were detected using respective antibodies described previously (21).

### Intracellular trafficking

Cells growing on coverslips in six-well plates were exposed to HPK or HSi at equivalent protein concentrations (20 μg/well) according to our previously established procedures (52). Briefly, cells were treated on ice for 1 h to promote receptor binding but not internalization, washed to remove unbound protein, and warmed to 37°C to promote synchronized uptake and intracellular trafficking. Cells were fixed at various time points after warming and then processed for immunofluorescence identification of HPK or HSi using an antibody that recognizes the polyhistidine tag (RGS-His antibody; Qiagen). Antibodies for RAB7 and EEA1 were purchased from Abcam (ab50533 and ab206860, respectively). Samples were imaged using a Leica SPE laser scanning confocal microscope. Acquired images were imported to ImageJ and split into individual channels. Individual cells in selected channels were delineated, and integrated densities were measured for each selected area.

### Immunocytofluorescence/immunohistofluorescence

Cells were fixed and processed for immunocytofluorescence, as previously described (52). Tissues harvested from mice were preserved in 4% paraformaldehyde in PBS and then transferred to 70% ethanol. Tissues were then paraffin embedded, sectioned, and mounted onto slides. The slides were deparaffinized by incubating in a dry oven for 1 h and then washing in xylene five times for 4 min each, followed by sequential rinses in 100%, 95%, 90%, 80% and 70% ethanol, twice each for 3 min. The slides were then submerged in water. Epitope retrieval was performed by incubating the slides for 30 min at 37°C in 20 μg/ml proteinase K in 10 mM Tris (pH 7.8). Following treatment, the slides were counterstained with 4',6-diamidino-2-phenylindole (DAPI; Thermo Fisher) and mounted with Prolong Antifade (Thermo Fisher). Images of the tissues were captured using a Leica SPE laser scanning confocal microscope. Images were analyzed using ImageJ.

### Subcellular fractionation

PB and HPK were internalized by MDA-MB-435 cells followed by biochemical isolation of cell compartments to assess the subcellular distribution of each protein. Specifically, cells were detached with 2 mM ethylenediaminetetraacetic acid (EDTA) in PBS (no trypsin to ensure that cell surface receptors for each protein remained unmodified) followed by 3–4 washes in PBS containing Ca^2+^ and Mg^2+^ to remove EDTA and resuspension of cells in buffer A containing ∼7 nmol PB or HPK (per 5 × 10^6^ cells). The mixtures were incubated with agitation for 2 h at 4°C to allow cell attachment but not internalization, followed by incubation for 2 h at 37°C to promote synchronized uptake. The cells were then pelleted, washed, and processed for subcellular fractionation using a Qproteome cell compartment assay kit (Qiagen), per the manufacturer's protocol.

### Native PAGE

HPK in storage buffer (20 mM HEPES [pH 7.45], 150 mM NaCl, 10% glycerol) was distributed into 150-μl aliquots at 1.33 μg/μl, and the pHs were adjusted by mixing with 350 μl of storage buffer at titrating pH levels for 30 min at 25°C or 37°C (0.4 μg/μl final protein concentration). Final pH values were confirmed using an Oakton pHTestr 50S (Cole-Parmer). Each sample underwent size filtration to retain HPK oligomers as previously established (21,44), and was sustained in its own pH buffer during application to spin columns that sufficiently retain HPK oligomers if present (Amicon Ultra 0.5-ml centrifugal filters, 100K NMWL). Each retentate (20 μl) was directly loaded on a Mini-protein TGX stain-free gel (4–15%; Bio-Rad); the 10% glycerol in the storage-incubation buffer was sufficient for loading the protein on the gel. After electrophoresis using standard native electrophoresis buffer (25 mM Tris, 192 mM glycine, pH 8.3) at 200 V for 35 min, the gels were visualized using a ChemiDoc MP imaging system (Bio-Rad). The bands were additionally visualized by Coomassie staining according to standard procedures.

### DLS

A Malvern ZEN 3600 Zetasizer Nano was used for DLS analyses. Each analysis comprised at least three measurements per sample, with each measurement comprising 100 runs at an average of 34k particle counts/s. The reported average is the number particle size determination parameter, which yields the most frequent particle size in the sample accounting for the intensity fluctuations of larger particles. The intensity of the particles was computed via Zetasizer software version 7.01, which applies the Stokes-Einstein equation to correlate the change in the scattering intensity and particle movements. For DLS of samples incubated at varied pHs, each pH buffer alone was first read to ensure proper signal-to-noise ratios. Noise below 100k counts/s at the lowest attenuation was considered acceptable to begin measurements.

### Electrophoretic mobility shift analysis (EMSA)

The ErbB2/HER2 synthetic siRNA duplex (50 pmol) was incubated with 0, 5 or 10 μg protein (PB, PBK or HPK) in 50 μl 0.1 M HEPES/Optimem I buffer for 20 min at RT and then subjected to electrophoresis on a 2% agarose gel (1:1, low-melting agarose/SeaPlaque GTG) in 0.5× Tris–borate–EDTA buffer. The gel was then stained with ethidium bromide to visualize siRNA and siRNA–protein complexes. Where indicated, isolated particles assembled as described in ‘Particle formation’ were incubated with heparin at various concentrations before gel electrophoresis.

### Isothermal calorimetry

Binding kinetics were determined using a Malvern Panalytical MicroCal PEAQ isothermal calorimeter, which assesses heat exchanges resulting from binding or dissociation by integrating the differential power that is required to maintain isothermal conditions. Matched buffer into buffer was assessed before each experiment to confirm that there was minimal heat exchange under baseline conditions before adding the analytes to the system. Two microliters of 20 μM siRNA solution was injected into 6 μM HPK at RT to yield the molar ratios displayed in the figures.

### Electron microscopy

Particles were prepared for transmission electron microscopy and imaged, as described previously (53), through the services of the Electron Imaging Center for NanoMachines within the California NanoSystems Institute at UCLA.

### Serum protection assay

Free siRNA alone (60 pmol) or preincubated with HPK (2 μg for 30 min at RT) was incubated in either complete medium (10% active bovine serum) or whole (100%) non-heat-inactivated serum at 37°C for 1 h and then assessed by agarose gel electrophoresis.

### Immunorecognition assays

ELISAs were used to determine the immunorecognition of HPK by anti-adenovirus serotype 5 (Ad5) antiserum compared to that for PB. HPK and PB were immobilized at 4 μg/well on an ELISA plate, and serum from Ad5-inoculated mice was added to each, followed by the detection of captured mouse Ig titers using standard procedures (44). A sandwich ELISA was used to compare the immunorecognition of HPK with that of HSi particles. Specifically, plates were precoated with rabbit anti-Ad5 polyclonal antiserum (ab6982; Abcam) and incubated in sodium bicarbonate coating buffer (pH 9.2), followed by a brief wash and blocking using 3% BSA. After washing with PBS, HPK and HSi particles were incubated overnight, followed by detection with a mouse anti-RGS-His antibody (34660; Qiagen) and anti-mouse horseradish peroxidase (A8924; Sigma-Aldrich). Colorimetric detection and absorbance measurements at *A*_650_ began 15 min after the addition of TMB (3,3′,5,5′-tetramethylbenzidine) substrate. The reaction was then stopped by adding 1 N HCl, and measurements at *A*_450_ were acquired.

### RNAi assays

To evaluate mRNA silencing *in vitro*, cells were plated in 96-well plates 36–48 h before treatment with various concentrations of HSi particles in complete medium (30–50 μl per well) with agitation at 37°C for 4 h and 5% CO_2_. Complete medium was then added to a total volume of ∼100 μl per well. For cells receiving siRNA lipoplexes, the siRNAs were assembled with Lipofectamine RNAiMax reagent (Thermo Fisher), and cells were treated 24 h after plating in a 96-well plate, according to the manufacturer's protocol. Plates were maintained at 37°C at 5% CO_2_ for 48–72 h after treatment, followed by cell lysis and the collection of RNA for quantitative PCR (qPCR; see below).

To evaluate ErbB2/HER2 protein knockdown by Western blotting, cells were treated with either siRNA lipoplexes or HPK-siRNA assemblies in complete medium and incubated for 48 h at 37°C, after which, the medium was exchanged for fresh medium. Ninety-six hours after transfection, ErbB2 levels were analyzed by western blotting. Lipoplexes were formed by incubating 100 pmol siRNA with Lipofectamine 2000 in Optimem I according to the manufacturer's protocol. HPK-siRNA complexes were formed by incubating 100 pmol siRNA with 20 μg HPK at a 1:2 or 1:4 (siRNA/protein) molar ratio in 100 mM HEPES in Optimem I buffer at RT for 20 min before adding to cells in complete medium. The cells were then lysed with RIPA buffer (150 mM NaCl, 50 mM Tris base [pH 8.0], 1 mM EDTA, 0.5% sodium deoxycholate, 1% NP-40, 0.1% sodium dodecyl sulfate, 1 mM dithiothreitol, 1 mM phenylmethylsulfonyl fluoride, and 1 mM Na_3_VO_4_) supplemented with complete protease inhibitor cocktail. Protein concentrations were determined using Bio-Rad protein assay dye reagent. The cell lysate proteins were separated by 10% PAGE (25 μg of total protein loaded per well), followed by electrotransfer (140 mA for 2 h) to a nitrocellulose membrane (Hybond-ECL; Amersham Biosciences, Piscataway, NJ, USA). The membranes were blocked in PBS containing 3% dry milk for 1 h at RT with constant agitation. The nitrocellulose was incubated with 1 μg/ml anti-ErbB2/HER2 (Upstate/Millipore, Billerica, MA, USA) diluted in PBS with milk, agitating at 4°C overnight. The membranes were washed twice with water and then incubated with secondary antibody for 1.5 h at RT. The membranes were washed twice with water and then with PBS with 0.05% Tween for 3–5 min. The nitrocellulose was rinsed with water 4–5 times and processed for chemiluminescence.

To evaluate ErbB2/HER2 protein knockdown by immunocytofluorescence, complexes containing HPK with lipoplexes were formed by incubating either 1.5, 5 or 10 μg of HPK with siRNA lipoplexes for a final molar ratio of 1:1:1, 1:2:1 or 1:4:1 (siRNA/HPK/Lipofectamine), respectively. These complexes were formed by incubating HPK with lipoplexes at RT for an additional 20 min after lipoplex formation, and then the complexes were added to the cells. Control cells were incubated in 100 mM HEPES/Optimem I buffer alone, with HPK only, or with siRNA only. Ninety-six hours after treatment, the cells were fixed and processed for immunocytofluorescence.

### qRT-PCR

RNA was collected with a Promega SV96 kit and complementary DNA (cDNA) was produced with a Bio-Rad iScript RT Supermix according to the manufacturers’ protocols. cDNA was stored in 10 mM Tris–HCl (pH 8.0), 0.1 mM EDTA buffer at −20°C until use. Ribogreen and SYBR green nucleic acid binding dyes were used to quantify RNA and cDNA, respectively. The qualities of RNA and cDNA samples were determined by measuring the *A*_260_/*A*_280_ ratios. Three samples per group were assayed, each containing 75 ng cDNA. A TaqMan gene expression assay was used to perform qPCR, and amplification products were analyzed using a CFX Connect qPCR system (Bio-Rad). The primer/probe sequences were as follows: RTF primer, TCTGGACGTGCCAGTGTGAA; RTR primer, TGCTCCCTGAGGACACATCA for *ERBB2* (accession number NG007503); luciferase probe set Mr03987587 (Thermo Fisher) for firefly luciferase (accession number AF093683). Unless otherwise indicated, data were analyzed using the ΔΔ*C_q_* method.

### Cytotoxicity assay

Cytotoxicity was measured by crystal violet staining, as previously described (53).

### *In vivo* procedures

All mice were obtained from Charles River Laboratories. All procedures involving mice were approved by the IACUC (protocol #4796) and were performed in accordance with the institutional and national guidelines for the care and use of laboratory animals. Data were collected in a single-blinded fashion, such that the identities of the treatment groups were unknown to the individual acquiring the measurements. For xenograft models, 6-week-old female immunodeficient (NU/NU) mice received bilateral xenograft implants of MDA-MB-435 tumor cells (1 × 10^7^ cells/implant). For immune-competent models, female 6-week-old BALB/c mice received bilateral mammary fat pad injections of 4T1-Luc cells (1 × 10^5^ cells/injection). For both models, mice were randomized at tumor establishment (≥100–150 mm^3^) into separate treatment groups (*n* = 5 mice per group). For whole-animal time-course imaging, mice received single tail vein injections of either siRNA or HSi (each equating 0.087 mg/kg Cy5-labeled synthetic siRNA, obtained from Invitrogen). The mice were viewed by multimode optical imaging to detect Cy5 fluorescence intensity, and images were acquired at various time points after injection. Images were acquired under constant gain settings to enable comparisons. The fluorescence contrast between tumor and nontumor regions was acquired by selecting specific known areas on the mouse images at the various time points and measuring the fluorescence intensities of each region. The contrast comparisons between tumor and nontumor regions in siRNA- and HSi-injected mice were obtained by subtracting the fluorescence intensities for each time point from the nontumor region from those at tumor regions for each mouse. To detect the biodistribution of near infrared (NIR)-labeled cargo, mice received single tail vein injections of the reagents equating 1.5 nmol Alexa Fluor 680-labeled siRNA and were imaged ∼4 h later using a Xenogen IVIS system, followed by tissue harvest and imaging of extracted tissue to acquire average radiance per tissue area. For therapeutic efficacy studies, the reagents were delivered by tail vein injections (0.087 mg/kg siRNA) twice weekly for 4–6 weeks, and tumor volumes (height × width × depth) were monitored ∼2–3 times/week under single-blinded conditions (treatment groups unknown to the individual acquiring the measurements). At the experiment's termination, all animals were sacrificed, and tissues were collected for follow-up analyses. To assess luciferase silencing *in vivo*, mice received a single tail vein injection (0.087 mg/kg siRNA/injection), and mRNA from the tumors was harvested ∼72 h later.

### Immunogenicity assay

Female BALB/c mice (∼6 weeks; Charles River) received tail vein injections of HSi-Scr siRNA at 1.5 nmol siRNA per injection once/week for 4 weeks. Dosages equated 0.5 mg/kg per injection of HPK. Replication-deficient Ad5 was delivered as a control inoculant at 1.2 × 10^9^ PFU/injection. Serum was collected and 1 × 10^−4^ dilutions were processed by ELISA, using either HSi-Scr (5 μg/ml, 0.5 μg/well) or Ad5 (5 × 10^6^ PFU/well) as capture antigens. Mouse Ig was detected using horseradish peroxidase-conjugated anti-mouse antibody, and ELISAs were performed according to standard procedures used elsewhere (44). Serial dilutions of mouse Ig were used as reference antibody titers.

### Statistical analyses

Except where indicated, statistical significance was determined by one-way analysis of variance followed by a Tukey's *post hoc* analysis. Statistical significance was set at 0.05. Error bars in figures represent standard deviations unless otherwise indicated. Sample sizes were determined by power analyses of our previous *in vitro* and *in vivo* data (54), which indicated that *in vitro* sample sizes have 80% power at the 0.05 significance level to detect a 0.18 difference between group means in a two-factor ANOVA, and *in vivo* sample sizes achieve at least 80% power at the 0.05 significance level in a repeated-measures ANOVA.

## RESULTS

### HPK forms HER3-binding capsomeres

**Figure 1.**
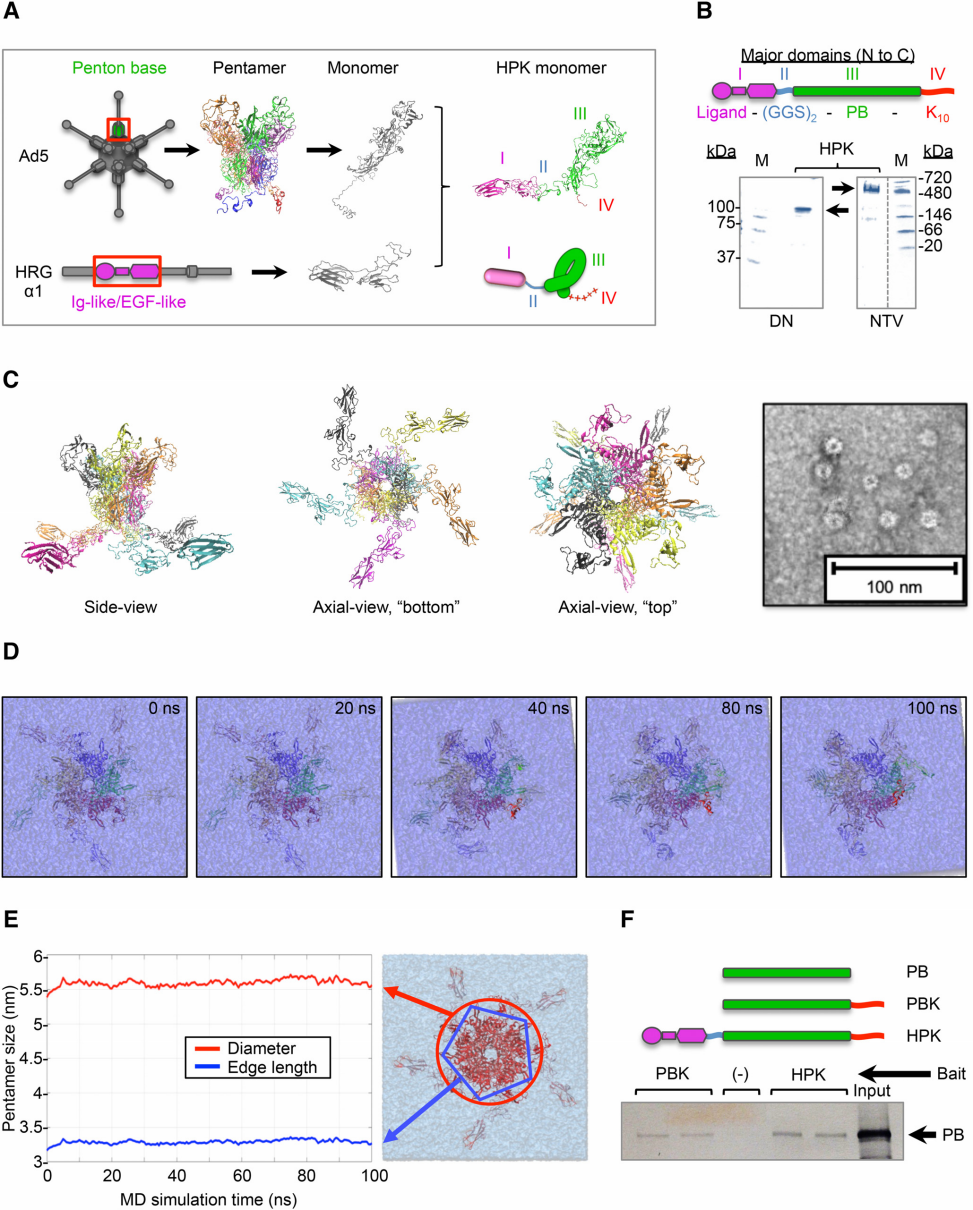
HPK forms soluble capsomeres with flexible ligands. (**A**) MD model of HPK monomer with functional domains delineated. Schematics show Ad5 capsid and heregulin proteins, with the PB pentamer (top) and receptor-binding domain of heregulin (bottom) in boxes. Ribbon structures of the corresponding polypeptide and folded chimera are shown. Each monomer of the PB and the functional domains of the HPK chimeric protein (assigned numerals I–IV) are delineated by different colors. Schematic below ribbon structure of the chimeric protein represents the folded monomer. (**B**) Expression of recombinant chimeric protein. Schematic shows the linear structure of HPK with functional domains delineated (from amino [N] to carboxy [C] terminus): I, targeting ligand; II, oligopeptide bridge composed of the neutral residues, Gly-Gly-Ser-Gly-Gly-Ser or (GGS)_2_; III, PB; IV, decalysine tail (K_10_). Protein gels show electrophoresis of HPK under denaturing (DN) and native (NTV) conditions. (**C**) MD structure of HPK pentamer. Ribbon model of HPK is shown as a pentamer with each monomer highlighted by a different color. Side and axial views (from the ‘top’ and ‘bottom’ of the pentamer) are shown (all sides of rotating pentamer can be viewed in Supplementary Movie S1). Transmission electron microscopy image of HPK preparation exhibiting self-assembled capsomere-like structures is shown on the right. EM image is contrast enhanced to highlight structure. (**D**) MD simulation of pentamer in solution. The images were captured at sequential time points of the MD simulation (the full simulation can be viewed in Supplementary Movie S2). (**E**) MD analysis of pentamer stability in solution. Two order parameters were tracked during MD simulations of the pentameric unit: (i) the diameter of the pentamer core (red) and (ii) the edge length of the pentameric projection of the core (blue). (**F**) Binding between PB-derived proteins. Blot shows labeled PB after co-precipitation with indicated bait protein prebound to nickel resin (shown in duplicates). Input, 5 μl of PB alone. (–) nickel resin lacking bait.

The PB serves as the foundational unit of HPK and thus provided the starting point for examining the architecture of HPK through computational structural modeling. In its natural state, the PB protein forms a homopentamer that normally caps each vertex of the Ad icosahedron (48) (Figure 1A). We previously constructed a recombinant gene encoding the Ad5 PB sequence fused to a carboxy-terminal decalysine (designated domain IV, for binding nucleic acids) (Figure 1A and B and Supplementary Figure S1A and C), producing the modified protein referenced here as PBK (55). Nondenaturing gel analysis has shown that PBK pentamerizes under native conditions (55).

The HER3-targeted version of this protein, designated HPK here, results from genetic modification of PBK to include an N-terminal ligand (domain I) derived from the minimal receptor-binding region of human heregulin (or neuregulin)-1α1 (56) (Figure 1A and B). The HPK gene construct includes a sequence encoding an oligopeptide linker (domain II) comprising neutral residues that separate the targeting ligand and PB (domain III) functional segments (Figure 1A and B) (56). Structural modeling of HPK under physiological conditions in solution suggests that the ligand and PB domains fold independently while remaining bridged together by an unstructured linker (Figure 1A).

Recombinant expression of HPK yields a product migrating at a molecular weight (MW) of between 90 and 100 kDa under denaturing conditions (Figure 1B) whose peptide identity has been verified through proteomics analysis. Under nondenaturing conditions, a product migrating between 480 and 720 kDa predominates (Figure 1B), suggesting that HPK oligomerizes in a native environment. In support, MD simulations show that HPK assumes a stable pentameric structure during a 100 ns relaxation under physiological conditions (Figure 1C). Pentamerization of HPK predicts a MW of ∼500 kDa, which closely aligns with the high-MW species seen with native gel electrophoresis (Figure 1B). The computer modeling of HPK yields a pinwheel-like configuration, with each targeting ligand extended in a splayed position from a central pentameric barrel formed by the PB domain of each HPK monomer (21) (Figure 1C and Movie S1). The central barrel is visible under transmission electron microscopy (Figure 1C) and resembles the ring-like configuration of viral capsomeres (57). The MD simulation of HPK suggests that pentamerization is stably maintained while the extended ligands have a flexible range of movement (Movie S2; image frames shown in Figure 1D). This observation is supported by measurements taken of both the outer diameter and pentamer edge length of the central barrel, which both show that the size is maintained during the 100 ns relaxation (Figure 1E). In further support, we found that HPK co-precipitated with soluble recombinant PB, suggesting that HPK oligomerizes through the PB domain (Figure 1F). Together, these findings suggest that the PB domain drives the stable self-assembly of HPK into pentamers.

**Figure 2.**
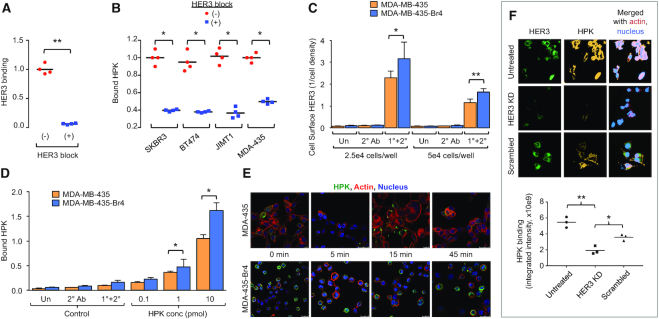
HER3-specificity. (**A**) ELISA showing binding of HPK to immobilized HER3 peptide; *y* axis reflects ELISA absorbance normalized by relative cell number, ‘HER3 block’ indicates HPK was preadsorbed with soluble HER3 peptide before incubating on the immobilized peptide. (**B**) Detection of HPK binding to HER3 on individual tumor lines by cell surface ELISA; ‘HER3 block’ indicates HPK was preadsorbed with a HER3 peptide at equimolar or 10-fold molar excess concentrations before incubation with cells. (**C–E**) HER3 expression, receptor binding, and particle uptake in brain metastatic cell line, MDA-MB-435Br4, in comparison to that in parental cells, MDA-MB-435. (**C**) Cell surface HER3 levels measured by cell surface ELISA. (**D**) Binding of HPK at indicated protein concentrations per well measured by cell surface ELISA. Un, untreated cells; Un, untreated cells; 2° Ab, secondary antibody alone; 1°+2°, primary and secondary antibodies. (**E**) Immunocytofluorescence and laser scanning confocal fluorescence microscopy of indicated cell lines at indicated time points of HPK uptake. Bars, 20 μm; *P< 0.05; **P< 0.01 (*n* = 3). **(F)** Effect of HER3 silencing on HPK binding to MDA-MB-435 cells. Micrographs show immunocytofluorescence of HPK and HER3 at ∼24 h after cells received HER3 or scrambled siRNA in comparison to untreated cells. Graph summarizes the relative levels of cell-bound HPK under HER3-silencing and control conditions. Each data point represents the average from three independent fields (>500 cells per field) in triplicate wells undergoing each corresponding (HER3 silencing or control) condition. KD, knockdown. *P< 0.05; **P< 0.01.

Our structural findings of HPK suggest that the ligands remain solvent exposed and thus should be available for receptor binding in a protein homo-oligomer. In agreement, HPK exhibits considerable binding to an immobilized peptide containing the extracellular domain of human HER3, which is blocked by preadsorption with the peptide *in vitro* (Figure 2A) and on HER3-expressing breast tumor cells (Figure 2B; cell surface HER3 levels shown in Supplementary Figure S2), confirming the receptor specificity of the ligand. In further support, HPK preferentially enters cells expressing high levels of HER3 versus those with low levels (Figure 2C–E). Specifically, the MDA-MB-435-Br4 (or Br4) cell line displays cell surface HER3 levels that are significantly higher than the parental line, regardless of cell density (Figure 2C). HPK exhibits concomitantly higher binding to the Br4 line than the parental line (Figure 2D), as well as measurably higher uptake at levels reflecting the differences in HER3 expression (Figure 2E). These findings were further validated by demonstrating that the silencing of HER3 in MDA-MB-435 cells significantly reduced the binding of HPK (Figure 2F).

### The HPK capsomere is endosomolytic and forms a pH-sensing barrel

**Figure 3.**
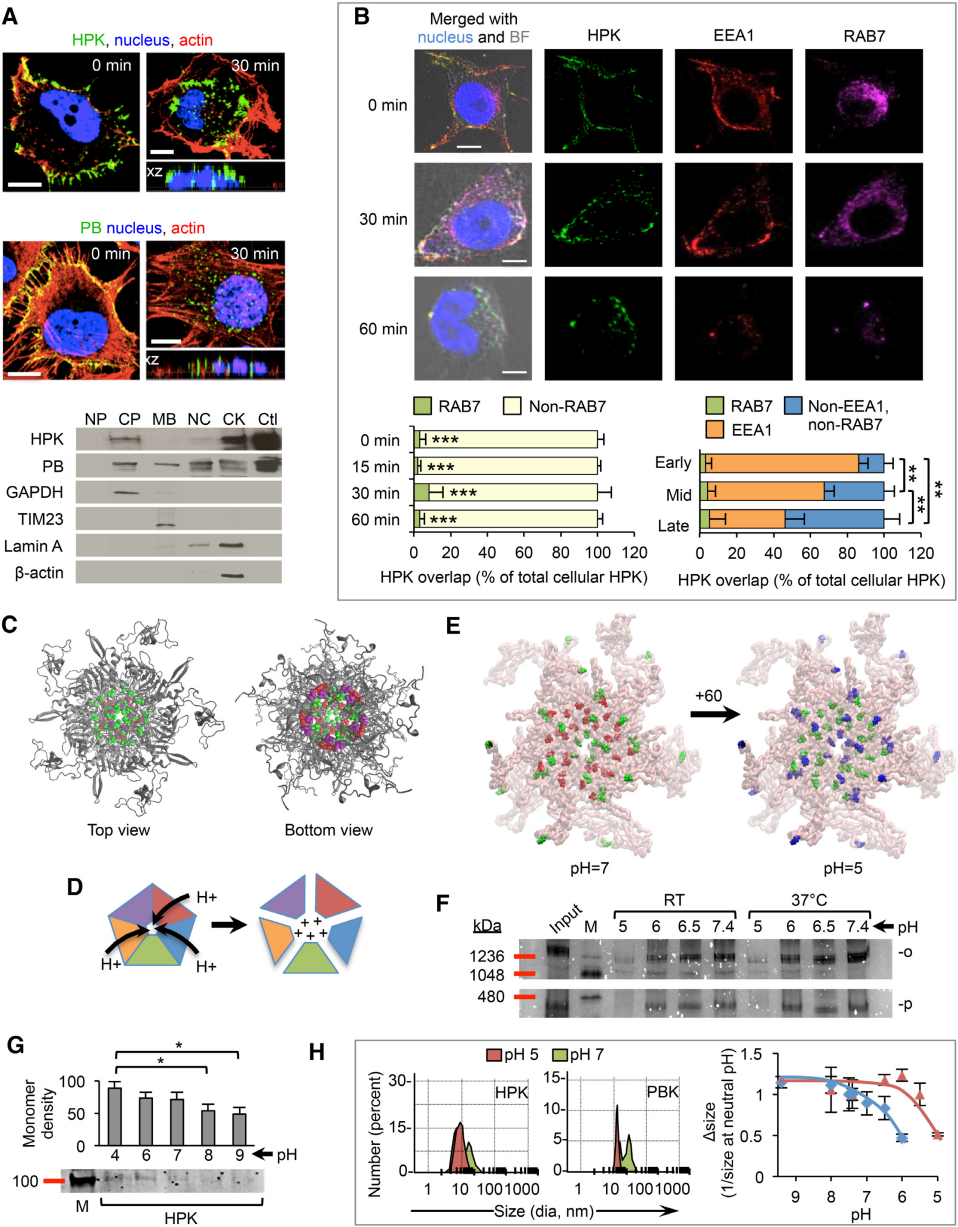
Cell entry. (**A**) Internalization and subcellular fractionation. Micrographs show intracellular trafficking of HPK or soluble recombinant PB after uptake in MDA-MB-435 cells. Side views of cells are shown in *xz* and *yz* planes. Bars, 5 μm. Western blots show subcellular fractions of MDA-MB-435 cells harvested 1–2 h after uptake probed with an antibody recognizing the PB. NP, no protein (untreated cell lysate); CP, cytoplasm; MB, membrane; NC, nucleus; CK, cytoskeleton; Ctl, control (protein alone, 5 μg). Fractionation controls delineating subcellular compartments include GAPDH (cytoplasmic), TIM23 (membrane), lamin A (nuclear and cytoskeletal), and β-actin (cytoskeletal). (**B**) HPK trafficking to early and late endosomes. Micrographs show the time-constrained trafficking of HPK (green) through initial binding (0 min), internalization (30 min), and intracellular transit (60 min). Early endosome marker EEA1 is shown (red) along with late endosome marker RAB7 (magenta). Left bar graph summarizes the percentage of internalized HPK overlapping with RAB7 at indicated time points (0, 15, 30 and 60 min) of uptake (*n* = 8 cells per time point) compared to the percentage of internalized HPK that does not overlap with RAB7 (non-RAB7). Right graph summarizes the percentage of internalized HPK overlapping with RAB7, EEA1, or neither (non-EEA1, non-RAB7) marker at early, mid, or late stages of cell entry. Stages are based on percentage of overlap with EEA1. **P< 0.01, comparing EEA1 overlap; ***P< 0.001, compared to non-RAB7. (**C**) Ribbon model of PBK. Titratable amino acids are represented by space-filling residues: purple, Lys/Arg; green, Asp/Glu; red, His. (**D**) Proposed capsomere dissociation in response to low pH. Schematic shows putative protonation of barrel interior and dissociation of monomers in response to acidification. (**E**) Structural modeling of HPK with titratable amino acids highlighted. The pH 7 structure shows the negatively charged Asp/Glu in red and neutral His residues in green. The pH 5 structure shows the neutral Asp/Glu residues in green and positively charged His residues in blue. Arrow indicates net gain of positive charges upon protonation, as summarized in Table 1. (**F**,**G**) Native PAGE of HPK after exposure to titrating pH and size retention. Protein gels show electrophoretic mobility of HPK before (input) and after size filtration to isolate retentates containing oligomeric protein. Samples were loaded onto gels in the same pH buffers in which HPK was incubated before and during filtration. The gels in panel F show HPK pentamers (p) and oligomers (o) postfiltration in comparison to the starting HPK protein (input) before aliquoting into pH-adjusted buffers and filtering to isolate species larger than monomers. The gel in panel G shows HPK monomers in nonfiltered samples after incubating under indicated pH conditions. Graph in panel G summarizes band densities. *P< 0.05; M, high-molecular-weight protein marker. (**H**) Effect of pH on average diameters of HPK and PBK. Histograms show sizes measured by DLS after exposure to pH 7 and pH 5 conditions described above. Graph summarizes average sizes measured by DLS after incubating in indicated pH titrations and represents at least three independent experiments.

Intracellular trafficking of HPK follows a similar pattern as that of wild-type PB (Figure 3A), which rapidly transits toward the nuclear periphery after cell entry (52,58), similar to the whole virus (59–61). Subcellular fractionation shows substantial entry of HPK into cytoplasmic and cytoskeletal compartments after uptake into HER3-expressing MDA-MB-435 cells (Figure 3A; relative cell surface HER3 levels shown in Supplementary Figure S2). These findings compare favorably with those for the wild-type PB, for which a considerable proportion is internalized to cytoskeletal and nuclear compartments (similarly to the trafficking of whole Ad) (59–61) that are otherwise inaccessible without endosomal disruption, while a small proportion was retained in the membrane fraction (Figure 3A). To investigate this further, we examined the intracellular trafficking of HPK and assessed whether it accumulated in early and/or late endosomes. Our findings reveal that during cell uptake, HPK transiently overlaps with EAA1-labeled early endosomes but not with RAB7-positive late endosomes-lysosomes (Figure 3B). Moreover, a diminishing localization of internalized HPK with early endosomes accompanied a concomitant increased distribution to non-EEA1 non-RAB7 compartments but no further accumulation in late endosomes-lysosomes (Figure 3B). These findings support the exit of HPK from early endosomes, which may be attributable to the PB domain (on the basis of its functional roles during Ad infection) (33,52,58); thus, we next investigated whether the capsomere structure contributes to endosomolysis.

Structural modeling of both PBK and HPK capsomeres revealed that the pentameric ring creates a solvent-accessible pore lined with His and other charged amino acids, specifically, negatively-charged glutamic (Glu) and aspartic (Asp) acids that appear to be counterbalanced by positively-charged lysine (Lys) and arginine (Arg) residues (Figure 3C and Movie S3). This observation led us to interrogate whether a low-pH environment such as that encountered in the endolysosome could lead to protonation of these residues and induce charge-mediated repellence of monomers (as depicted in Figure 3D), thus unmasking hydrophobic domains mediating pentamerization (48). On the basis of the structural models, a pentameric configuration has a buried surface area of 41 413 Å^2^, of which 19 133 Å^2^ is hydrophobic (Supplementary Figure S3) and will be exposed when the pentameric structure breaks into its constituent components. When exposed, these domains could interact with membrane lipids and destabilize the endolysosomal membrane, enabling the penetration of both the PB and HPK into soluble cell compartments, as observed earlier (Figure 3A). To examine this, we first assessed the protonation states of all titratable amino acids in the capsomere barrel of both PBK and HPK using the PropKa server (50). At a physiological pH of ∼7.0, the Glu and Asp residues are negatively charged (except five Asp residues, due to their local environment) and all His residues are neutral, resulting in a net −10 charge on the PBK pentamer (Table 1). At an acidic pH of 5.0 (as encountered in endolysosomes), 30 of the 430 acidic residues (Asp/Glu) and 30 of the 50 His residues become protonated, leading to some neutral Glu and Asp residues and many positively charged His residues (Table 1). Hence, a transition from pH 7.0 to pH 5.0 would yield a net +55 change in charge for the PBK pentamer (Table 1). The stability of the HPK pentamer shown in the MD simulations (Movie S2) indicates that the titratable residues identified in the PBK pentamer barrel are retained in HPK capsomeres. Accordingly, these same residues would undergo charge conversion in the HPK pentamer upon a change in pH from 7.0 to 5.0 (Figure 3E), while additional residues introduced by the targeting ligand contribute to an overall pH-mediated +60 shift in charge (Figure 3E and Table 1).

**Table 1.** Protonation states of titratable amino acids

	PBK		HPK	
pH	7	5	7	5
Arg (+1)	175	175	215	215
Lys (+1)	145	145	250	250
His (+1)	0	30	0	30
His (0)	50	20	55	25
Asp (0)	5	15	0	10
Asp (-1)	170	160	190	180
Glu (0)	0	15	0	20
Glu (-1)	160	145	260	240
Pentamer charge	-10	45	15	75
Δ charge, pentamer	+55		+60	

The location of these charges within the pentamer barrel is likely to cause strong positive charge-mediated repellence of the protein monomers. To test this, we examined whether HPK undergoes a size shift from oligomers to monomers under acidifying conditions. Nondenaturing gel electrophoresis of HPK under neutral conditions showed the presence of pentamers and higher MW oligomers (Figure 3F, ‘input’). Size filtration of HPK under defined pH conditions before electrophoresis enabled us to examine whether these species were retained with increasing acidification. Whereas pH 7.4 had no effect, reducing the pH to 5.0 dramatically reduced the presence of pentamers and oligomers (Figure 3F, ‘5.0’), with a concomitant increase in monomers (Figure 3G). In further support of this, DLS measurements showed that a shift from neutral to acidic pH shifted the size of HPK (as well as PBK) to a smaller species (Figure 3H). The additional +5 net positive charge that would occur upon protonation of HPK (compared to PBK) (Table 1) suggests that HPK may possess a higher sensitivity to pH conditions than PBK. In agreement, a broader set of pH increments enabled us to observe that HPK exhibits a reduction in size at a milder acidity (∼pH 6) compared to that for PBK (∼pH 5) (Figure 3H).

### HPK capsomeres assemble with siRNA into serum-stable multivalent nucleocapsids

**Figure 4.**
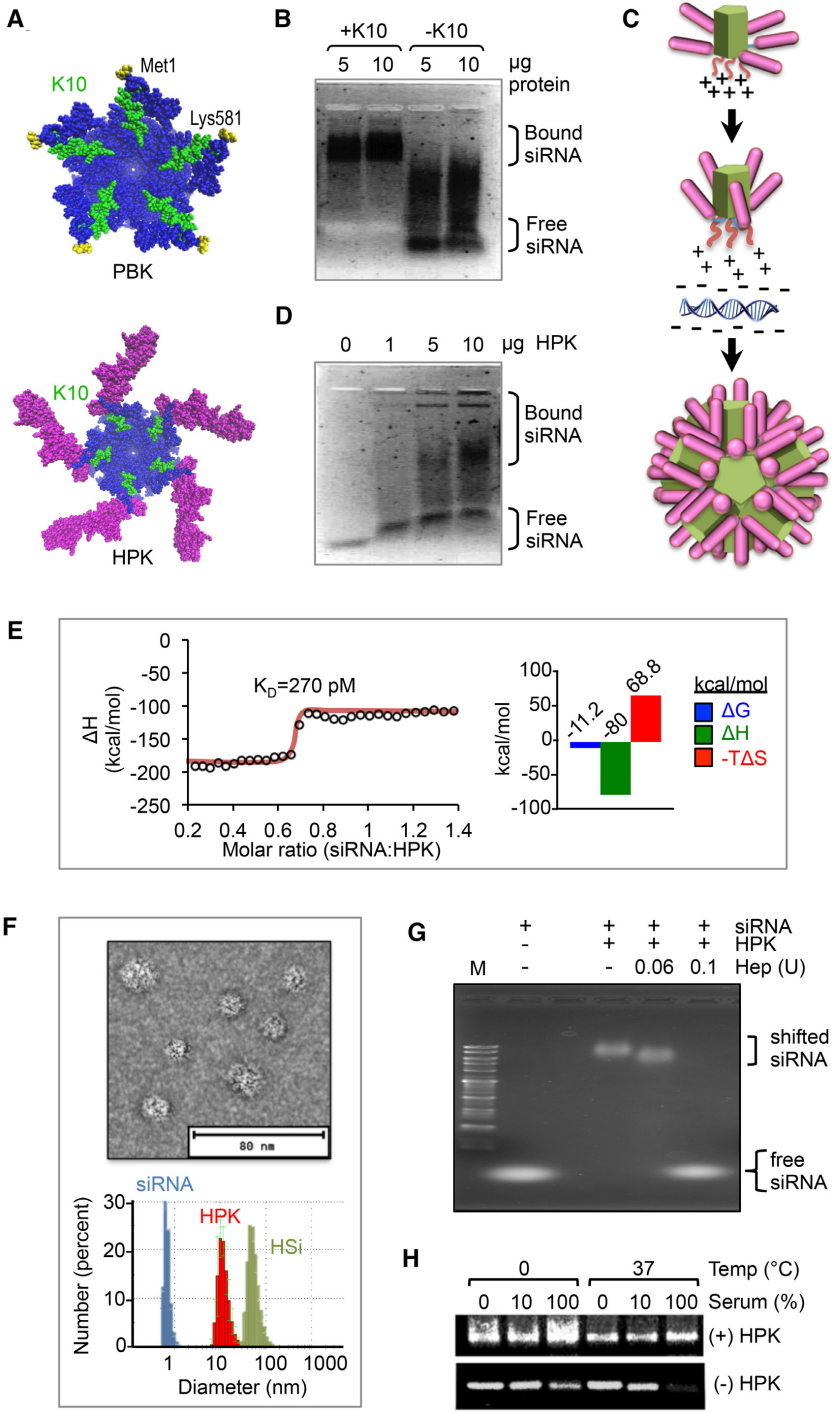
Assembly with siRNA into serum-stable nucleocapsids. (**A**) Space-filling models of PBK and HPK pentamers, showing decalysine sequences (highlighted in green). MD structures show each pentamer viewed from the ‘bottom.’ The PBK C-terminal residues (Lys581) are indicated. The N-terminal residues (Met1) are in yellow. The HPK targeting ligands in magenta. (**B** and **D**) EMSAs of siRNA after incubation with HPK, PBK, or PB at indicated concentrations. Figure shows an inverse image of ethidium bromide-stained gel. (**C**) Schematic of HPK pentamer undergoing hypothetical conformational changes to accommodate siRNA packaging. Protein domains are colored corresponding to the structure shown in panel A. (**E**) Binding kinetics of HPK with siRNA. Binding curve reflects heats recorded by isothermal calorimetry during titration of siRNA into HPK. Recorded heats were integrated to derive Δ*H* and *K_d_* values. (**F**) Particle shape and size. Panels show transmission electron microscopy of HSi particles (top) and average hydrodynamic diameter of HSi particles in comparison to individual components (siRNA, HPK) measured by DLS (bottom). EM images were contrast enhanced to highlight structure. (**G**) EMSA after ultrafiltration of complexes. Ethidium bromide-stained agarose gel shows relative electrophoretic migration of free siRNA or HSi particles (∼500 ng siRNA per lane) after isolation by ultrafiltration. (**H**) Stability of HSi in active serum. Electrophoresis and ethidium bromide staining of siRNA after incubating in active (non-heat-inactivated) serum under the indicated conditions followed by gel electrophoresis. (–) or (+) HPK, lacking or including siRNA preincubation with HPK, respectively, before exposure to serum.

Our structural modeling shows that pentamerization of both HPK and PBK places the polylysine domains of each monomer at a single face on the pentamer (Figure 4A), forming a cation-rich surface that should interact strongly with anionic molecules such as nucleic acids. In support, the electrophoretic mobility of siRNA is reduced by the decalysine-containing protein (+K_10_) in contrast to corresponding protein lacking the decalysine sequence (-K_10_) (Figure 4B). The structure of HPK suggests that its binding to siRNA requires ligand bending toward the PB barrel to accommodate protein packing into particles (Figure 4C), which may yield intermediate species of siRNA-bound protein reflecting multiple conformations during particle assembly. In agreement, titration of HPK onto siRNA results in multiple electrophoretic species of bound siRNA, with the higher MW species becoming more prominent as protein concentration—and hence particle packing—increases (Figure 4D). These findings are supported by isothermal calorimetry measurements showing that interactions between HPK and siRNA are saturable, become less disordered with increasing titration, and occur at a subnanomolar binding affinity (*K*_d_ = 270 pM) (Figure 4E).

Isolated complexes exceeding ∼10 nm in diameter yield spherical particles with hydrodynamic diameters ranging between 40 and 60 nm (Figure 4F) that constitute a single electrophoretic species (Figure 4G). Additional DLS parameters based on intensity- and volume-weighted distributions corroborate these findings, and larger species that would be indicative of aggregates in solution were not detected (Supplementary Figure S4A). Exposure to heparin to mimic the anionic environment of the cytoplasm (62) facilitated the release of the siRNA payload (Figure 4G) and enabled the quantification of protein and siRNA content. Here, heparin-mediated disassembly yielded a protein/siRNA molar ratio of (4.5–5.5):1, suggesting a stoichiometry of one siRNA molecule per pentameric unit (Table 2). Together, these findings support a model in which the siRNA 21-mer (which bears a net charge of −44) can neutralize the five K10 tails (net charge of +50) of the HPK pentamer [(HPK)_5_], thus preventing electrostatic repulsion between capsomeres and allowing them to converge into a spherical particle based on the shape complementarity of the pentamer units inherited from the Ad5 viral protein (shown in Supplementary Figure S4B). We furthermore found through computational structural modeling (Supplementary Figure S4B) that these intracapsomere contacts are dominated by polar interactions (hydrogen bonds and salt bridges) upon charge neutralization of the K10 tails by the siRNA. Whereas the minimum composition of such a sphere would equate to at least 12 (HPK)_5_ units [(HPK)_60_], predicting a diameter of ∼30 nm based on the atomic structure of HPK (see Supplementary Methods), the ability to accommodate the siRNA cargo may require additional pentamers to widen the capsule, thus yielding a slightly larger diameter, in agreement with our DLS measurements (Supplementary Figure S4). The resulting particles—designated HSi—resist destabilization in serum: exposure of naked siRNA to active serum results in rapid degradation by serum nucleases, whereas assembly with HPK protects the siRNA from serum-mediated degradation (Figure 4H).

**Table 2.** Protein:siRNA stoichiometry of HSi particles^a^

	Samples^b^			
	1	2	3	4
siRNA conc (ng/μl)	99.01	105.22	94.30	121.30
HPK conc (μg/μl)	3.1	3.8	3.6	4.1
siRNA (pmol/μl) ^c^	7.44	7.91	7.09	9.12
HPK (pmol/μl) ^d^	33.70	41.30	39.13	44.57
Molar ratio (HPK:siRNA)	4.5	5.2	5.5	4.9

^a^Particles were assembled and filtered to isolate high MW complexes as described in the Methods. To measure siRNA content, particles were disassembled with heparin followed by exposure to a nucleic acid binding dye (RiboGreen; Thermo Fisher Scientific) and extrapolation of fluorescence measurements against a standard curve of known siRNA concentrations. Protein content was measured by spectrophotometric absorbance using the Bradford method (Bio-Rad dye assay).

^b^Samples and corresponding information on protein and siRNA content were randomly selected from logged stocks of prepared particles.

^c^Based on approximate MW of 13 300 g/mol.

^d^Based on approximate MW of 92 000 g/mol.

**Figure 5.**
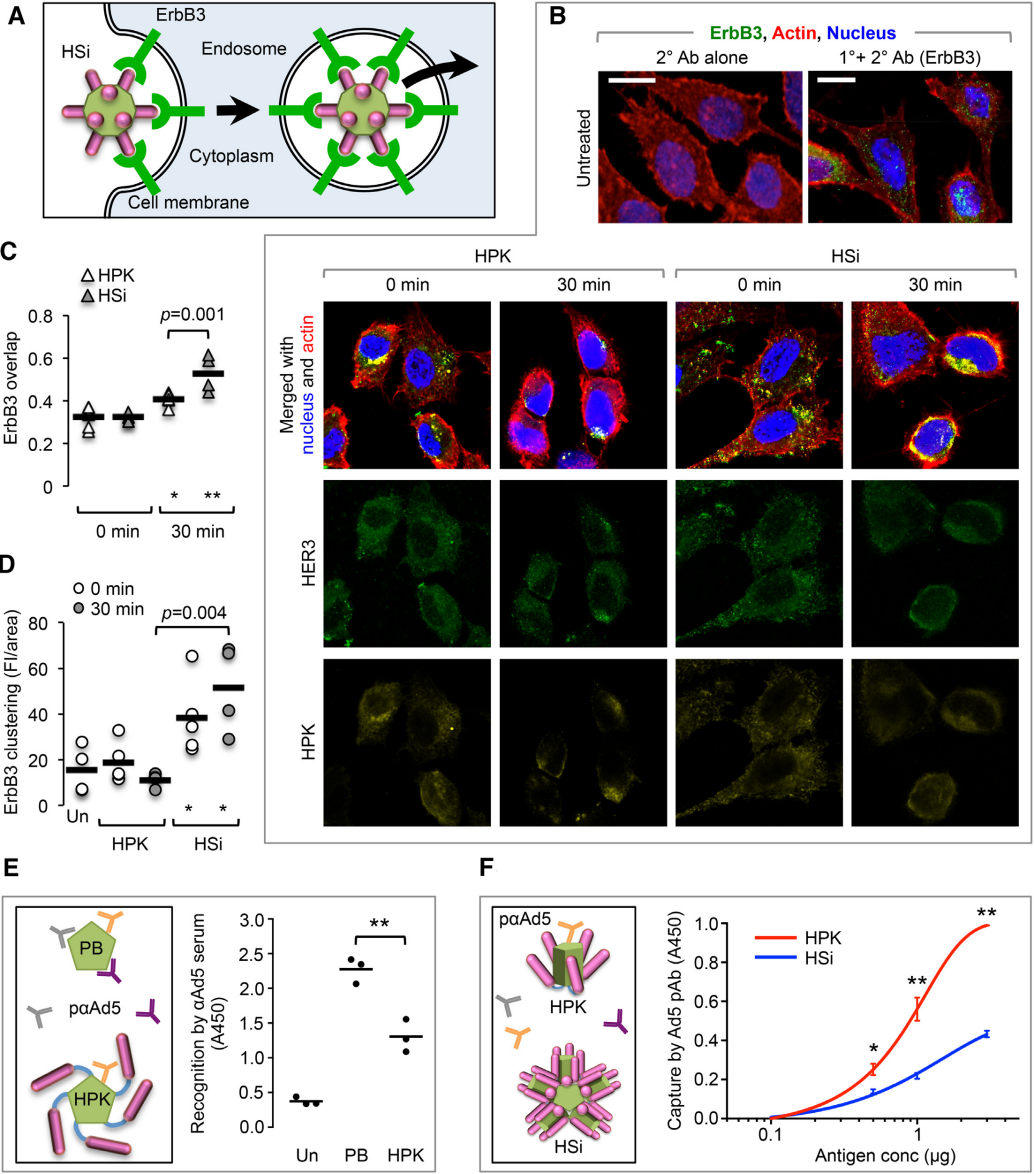
Contribution of ligand multivalency to cell interactions and immune shielding. (**A**) Multivalent interaction with HER3. Schematic illustrates how the multivalency of HSi may induce receptor clustering in cells. (**B**) Spatiotemporal localization of HER3 (ErbB3) during HPK or HSi uptake in MDA-MB-435 cells, shown by laser scanning confocal microscopy of cells fixed after indicated treatment and processed for immunocytofluorescence. Bars, 10 μm. (**C**) Ratios of HER3 overlap with HPK or HSi to total fluorescence at the indicated time points during cell uptake. *P< 0.05; **P< 0.01 versus respective 0-min timepoint (*n* = 4). (**D**) Quantification of the HER3 fluorescence intensity. Bars are the mean integrated densities of fluorescence for HER3 and symbols show individual data points for each treatment. Un, untreated cells. *P< 0.05 versus Un (*n* = 3). (E, F) Immunorecognition by anti-Ad5 capsid antiserum. (**E**) Schematic of antibody interactions with PB versus HPK (left). ELISA probed with Ad5 antiserum using PB and HPK as antigens (right). (**F**) Schematic comparing HPK and particle (HSi) interactions with Ad5 antiserum (left). ELISA probed with Ad5 antiserum using HPK and HSi as antigens (right). *P< 0.05; **P< 0.01 (*n* = 3).

Taken together, these findings suggest that siRNA becomes encapsulated, or encapsidated, by HPK upon HSi assembly, yielding a particle with multivalent ligands. To evaluate this functionally, we assessed whether HSi recruited higher levels of HER3 than HPK alone upon tumor cell binding and uptake, as depicted in Figure 5A. We observed that both HSi and HPK caused significant subcellular reorganization of HER3 upon cell binding, resulting in localized areas of HER3 convergence compared to that in untreated cells (Figure 5B). Importantly, the initial receptor binding was performed at a low temperature to promote ligation but not internalization, thus preventing or stalling energy-dependent processes, such as the translation of new HER3 protein. During cell uptake, the overlap of both HPK and HSi with HER3 increased compared to initial receptor binding (30 min vs 0 min, Figure 5C), which might have resulted from an increased recruitment of receptors (including nonligated receptors) to areas of internalizing receptor-ligand complexes. However, HER3 showed significantly higher overlap with HSi than with HPK during cell uptake (Figure 5C), corresponding to augmented HER3 clustering induced by HSi compared to that by HPK (Figure 5D). Altogether, these findings show that HSi influences HER3 distribution to a greater extent than HPK alone upon cell interaction, supporting a particle structure bearing ligand multivalency.

We also assessed the impact of ligand multivalency on immune recognition. On the basis of the MD simulation of HPK, the ligands are in a solvent-exposed position on the capsomere (Movie S2) that may partially mask the PB domain from immune recognition (Figure 5E, schematic). In agreement, immunorecognition by polyclonal anti-Ad5 antiserum was significantly lower for HPK than for PB (Figure 5E). Assembly of the HPK capsomere into HSi particles should add further ligand multivalency to the resulting structure and presumably further hinder immunorecognition of the PB (Figure 5F, schematic). In agreement, immunorecognition by polyclonal anti-Ad5 antiserum was lower for assembled particles (HSi) than for capsomeres (HPK) (Figure 5F). This same antiserum otherwise recognizes the PB domain under denaturing conditions (52,55,56) (Supplementary Figure S1B).

### HPK nucleocapsids facilitate HER3-directed siRNA delivery *in vivo*

**Figure 6.**
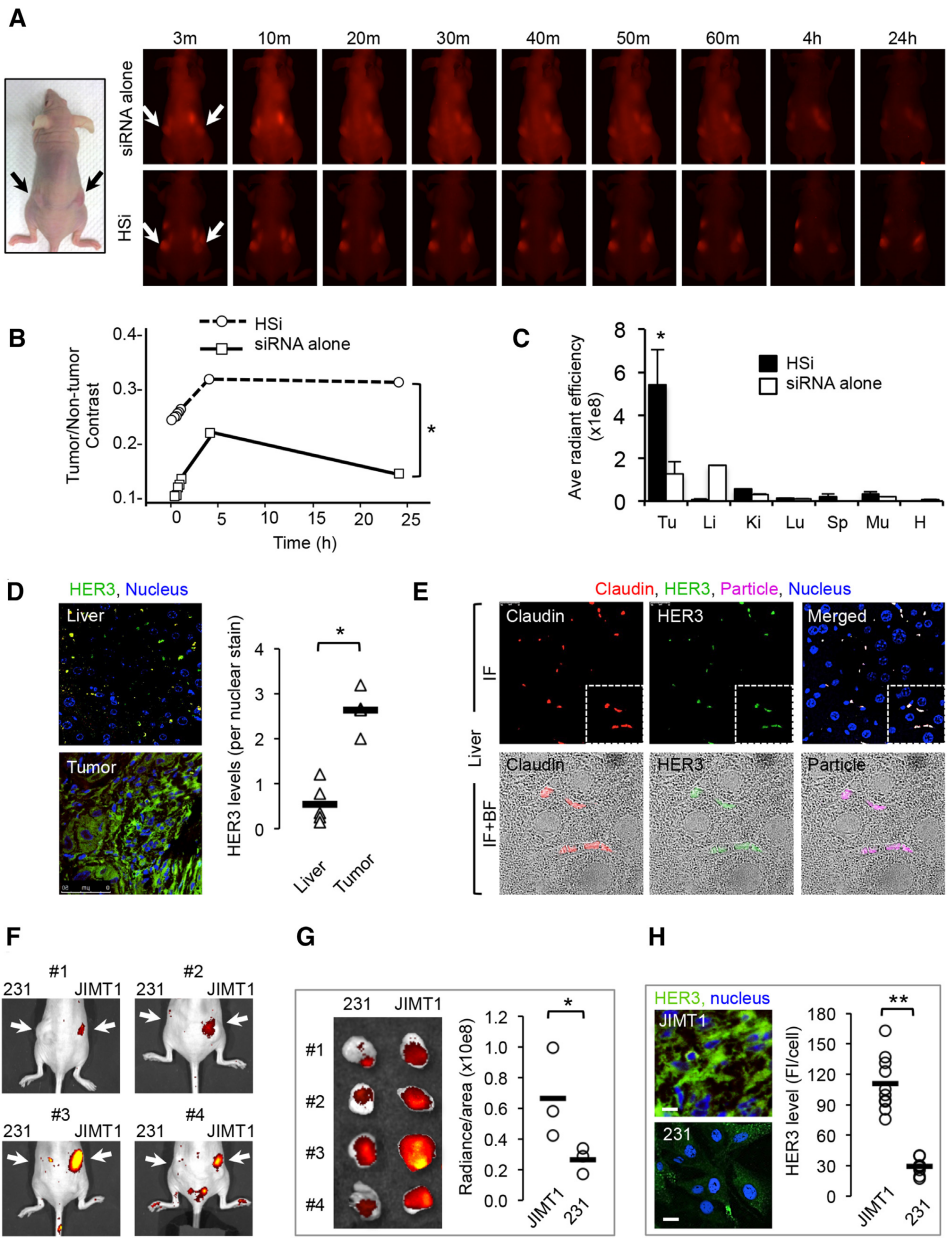
Biodistribution and tumor targeting. (**A**) Live animal imaging of mice bearing bilateral MDA-MB-435 tumors captured at sequential time points after single tail vein injections of Cy5-HSi or free Cy5-siRNA (each equating 0.087 mg/kg Cy5-labeled synthetic siRNA). Schematic shows orientation of images, with tumors indicated by arrows. (**B**) Fluorescence contrast comparisons between tumor and nontumor regions of mice injected with siRNA alone versus HSi from panel A. *P< 0.05 at all time points (*n* = 10). (**C**) Quantification of HSi tissue distributions compared with that of siRNA alone 24 h after single tail vein injections. *, *P*<0.05 versus all other samples (*n* = 3). Tu, tumor; Li, liver; Ki, kidney; Lu, lung; Sp, spleen; Mu, muscle; H, heart. (D, E) Comparison of tumor versus liver distributions after systemic particle delivery. (**D**) Immunohistofluorescence images showing HER3 staining (green) of tumor and liver specimens from the same tumor-bearing mice ∼2.5 h after systemic particle delivery (blue, nuclei). Graph summarizes the quantified differences in HER3 levels between tumor and liver tissues. *P< 0.05 (*n* = 5). (**E**) Higher magnification of liver specimens showing labeled particle in comparison to HER3 and claudin 5 staining. Lower panels show magnification of delineated region in upper panels. IF, immunofluorescence; BF, brightfield. (**F**) Tumor distribution in mice bearing both HER3-high (JIMT1) and HER3-low (231) tumors. Images show NIR signal acquisition from mice after receiving single tail vein injections of particles. (**G**) Imaging of harvested tumors from mice shown in panel F. Graph shows the quantification of NIR-labeled particles in each tumor. (**H**) Immunohistofluorescence imaging and quantification of HER3 levels on respective tumor types. *P< 0.05; **P< 0.01 (*n* = 4).

HPK showed improved siRNA delivery to tumors *in vivo* when we compared the spatiotemporal distribution of systemic HSi to that of naked siRNA labeled with Cy5 (Figure 6A–C). As shown in Figure 4H, naked siRNA was quickly degraded in serum, whereas HPK afforded protection from serum nucleases; hence, the fluorescence signal measured from naked siRNA may reflect the Cy5 label liberated from degraded siRNA. In comparison, the Cy5 distribution after HSi delivery exhibited higher tumor-to-nontumor contrast at each time point (Figure 6B) and higher tumor retention than naked siRNA at 24 h after injection (Figure 6A and C). Naked siRNA delivery resulted in similar Cy5 distributions in the liver and tumors, whereas HSi accumulated in tumors at a level that was >10-fold higher than in the liver and other nontumor tissue (Figure 6C). A closer look at the tissues showed that HER3 levels were considerably lower in the livers, where it was sparsely distributed, than in tumors (Figure 6D). At higher magnification, HER3 showed strong overlap with claudin 5, which delineates tight junctions in the liver (Figure 6E). The few liver-associated particles that were detected were restricted to these sites, with little to no observable particle entry into the liver parenchyma (Figure 6E).

To further evaluate the tumor-homing capacity of HPK nucleocapsids *in vivo*, we assessed delivery in mice bearing two different human breast tumors with high and low/no HER3 expression (JIMT1 and 231, respectively) (Figure 6F). Systemically delivered nucleocapsids preferentially accumulated (2–3-fold) in JIMT1 compared to 231 tumors (Figure 6G), corresponding to the 2–3-fold differences in HER3 levels (Figure 6H).

### HPK targets RNAi to HER3-expressing tumor cells

**Figure 7.**
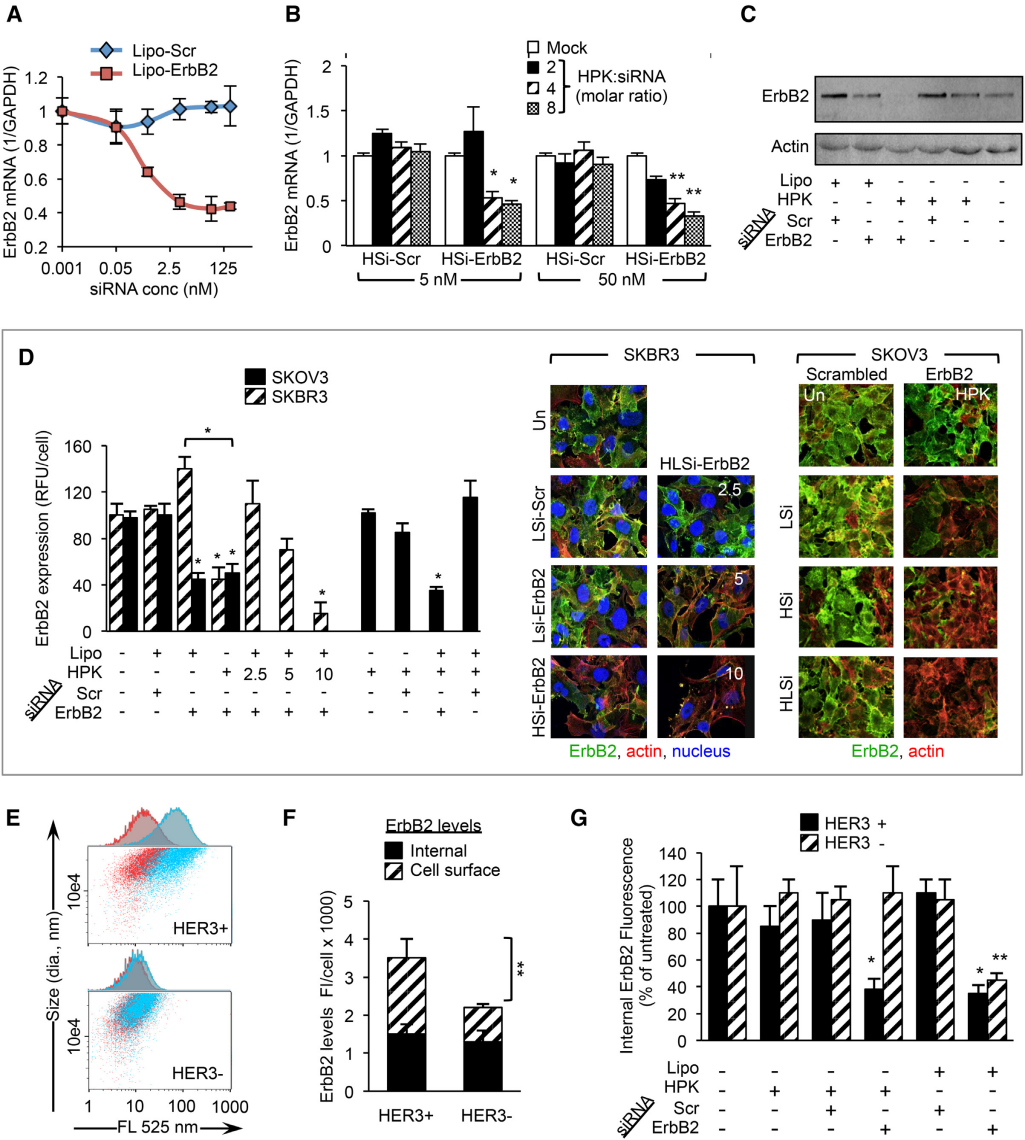
Comparison with commercial transfection. (A, B) qPCR of *ERBB2* mRNA isolated from MDA-MB-435 cells after *in vitro* treatments with cationic liposomes (**A**) or HSi (**B**) delivering *ERBB2* or scrambled siRNA. *P< 0.05; **P< 0.01 versus corresponding HSi-Scr treatments (*n* = 4). (**C**) Western blotting (anti-ErbB2 and anti-actin) of MDA-MB-435 cell lysates collected 96 h after indicated treatments. Lipo, Lipofectamine; siRNA-Scr, scrambled siRNA; siRNA-ErbB2, anti-ErbB2 siRNA. (**D**) Immunocytofluorescence of ErbB2 protein in SKBR3 human breast cancer cells or SKOV3 human ovarian cancer cells after delivery of ErbB2 or scrambled (Scr) siRNA by lipoplexes or targeted complexes. LSi, lipoplexed siRNA; HLSi, lipoplexed siRNA with 2.5, 5 or 10 μg HPK added equating 1:1:1, 1:2:1 or 1:4:1 siRNA/HPK/liposome, respectively. The 1:2:1 ratio is shown for SKOV3. Graph summarizes quantification of ErbB2 immunocytofluorescence normalized per cell. *P< 0.05 versus respective untreated cells unless otherwise indicated (*n* = 4). (**E**) Flow cytometry of MDA-MB-435 (top) and MDA-MB-231 (bottom) tumor cells based on cell surface HER3 levels. Red, untreated; blue, αHER3 antibody treated. (**F**) Comparison of ErbB2 immunocytofluorescence in MDA-MB-231 (HER3^−^) and MDA-MB-435 (HER3^+^) cells, showing proportions of internal and cell surface ErbB2. **P< 0.01 (*n* = 4). (**G**) Comparison of ErbB2 knockdown between HER3^+^ (MDA-MB-435) and HER3^−^ (MDA-MB-231) cells. ErbB2 levels are expressed as internal ErbB2 immunocytofluorescence normalized by cell number. *P< 0.05; **P< 0.01, versus untreated cells (*n* = 4).

To examine how HPK affects siRNA efficacy, we evaluated the gene-silencing activity of siRNAs delivered by HSi in comparison to commercial lipofection. As ErbB2 knockdown can induce apoptotic tumor cell death (63,64), we used a siRNA against the ErbB2/HER2 tyrosine kinase in several human tumor cell lines expressing both HER2 and HER3. We first examined the efficacy of mRNA knockdown with siRNAs delivered nonspecifically by cationic liposomes. Importantly, mRNA was extracted for qPCR before cells exhibited signs of cytotoxicity. The minimum siRNA concentration eliciting maximum ErbB2 mRNA reduction via lipoplex delivery occurred at ∼5 nM (Figure 7A). Similarly, HSi-mediated delivery of ErbB2 siRNA (HSi-ErbB2) significantly reduced ErbB2 mRNA levels compared to HSi-mediated delivery of a scrambled siRNA sequence (HSi-Scr) at HPK/siRNA molar ratios of at least 4:1 (Figure 7B); a lower molar ratio was not as effective. Importantly, HSi performed better than commercial lipofection under physiological conditions mimicking systemic delivery (i.e. in complete medium at 37°C with constant agitation) (Figure 7C). HSi substantially reduced ErbB2 protein levels in several types of human tumor cell lines, including those of breast, ovarian, and melanoma origins (Figure 7D). HPK also augmented lipofection-mediated delivery of ErbB2 siRNA, further reducing ErbB2 protein levels in comparison to lipofection alone (Figure 7D). We also compared human tumor-derived cells with high (HER3^+^ MDA-MB-435 cells) or low/undetectable (HER3^−^ MDA-MB-231 cells) cell surface HER3 expression but with comparable levels of ErbB2 in the cell cytoplasm (Figure 7E and F). Commercial lipofection reduced ErbB2 similarly in both tumor lines, whereas HSi reduced ErbB2 only in the HER3^+^ cells (Figure 7G), demonstrating the specificity of HSi-mediated delivery.

**Figure 8.**
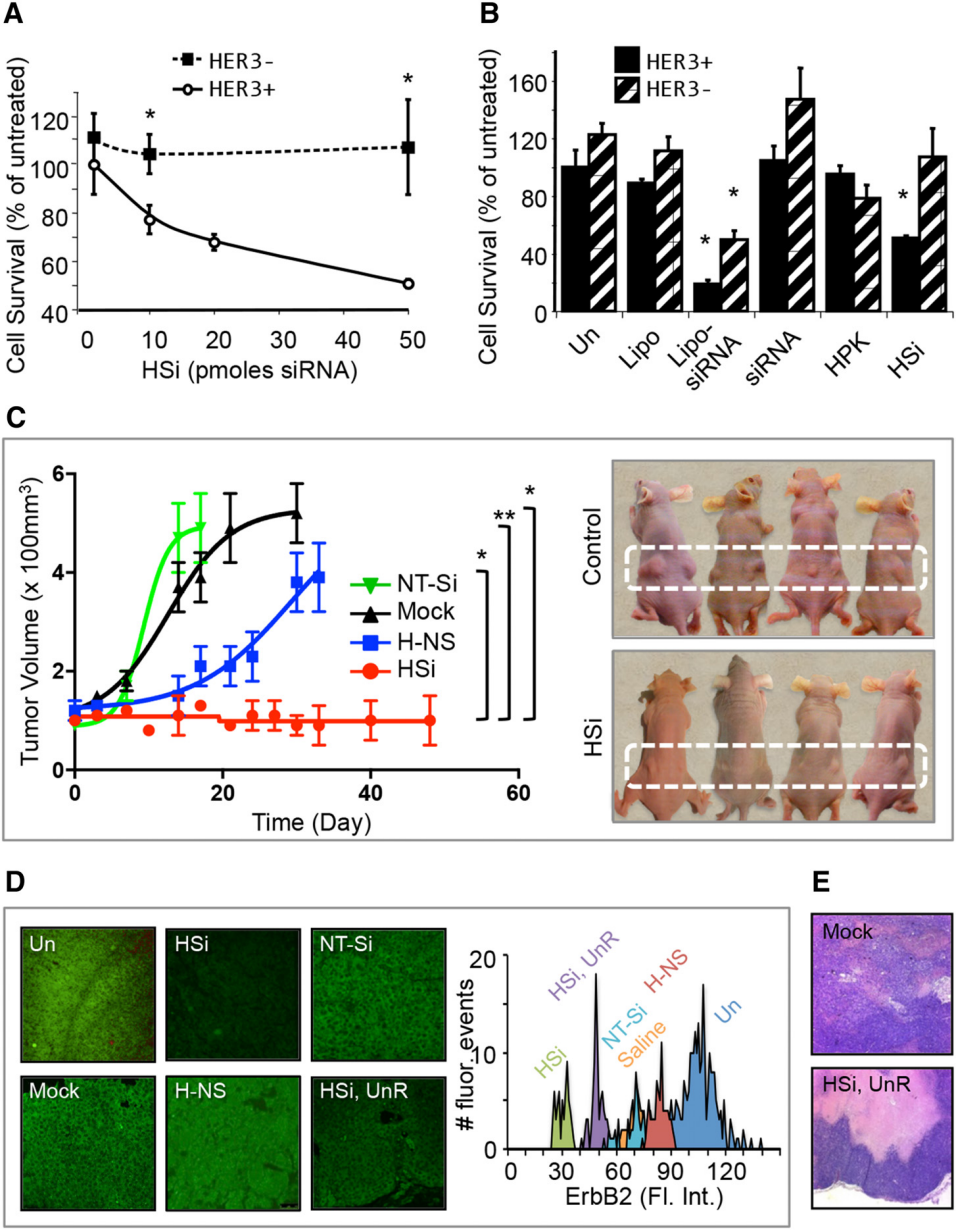
Delivery in ErbB2 xenograft model. (A, B) Survival of HER3^+^ (MDA-MB-435) and HER3^−^ (MDA-MB-231) tumor cells after *in vitro* exposure to titrating concentrations of HSi-ErbB2 (**A**) or liposomes alone (Lipo), lipoplexed siRNA (Lipo-siRNA), siRNA alone, HPK alone, or HSi, each given in a single treatment (**B**). *P< 0.05 versus corresponding untreated (Un) cells (*n* = 3). (**C**) Growth rates of MDA-MB-435 xenograft tumors in mice receiving tail vein injections of HSi (delivering ErbB2 siRNA, equating 0.087 mg/kg siRNA per injection) or indicated controls. Mock, saline injected; H-NS, nonsilencing particles; NT-Si, nontargeted siRNA. *P< 0.05; **P< 0.01 (*n* = 5). Significances refer to end-point tumor volumes at the time of sacrifice. Images show representative control (mock) and HSi-treated mice at the time of sacrifice for comparison of the tumor sizes. (**D**) ErbB2 immunohistofluorescence (green) of the tumor tissue extracted from mice in panel C. Histograms show quantifications of ErbB2 immunohistofluorescence intensity. (**E**) Hematoxylin and eosin staining of tumor specimens from control and HSi-treated mice. HSi unresp, tissue from tumor that appeared unresponsive (based on tumor volume) to HSi treatment.

To further validate the efficacy of HSi, we measured the survival of these cells treated with various amounts of siRNA. The survival of HER3^+^ but not HER3^−^ tumor cells was reduced by HSi in a concentration-dependent manner (Figure 8A). By contrast, the survival of both cell types was reduced by siRNAs delivered by commercial lipofection (Figure 8B). Intravenous delivery (via tail vein) of HSi in mice bearing human xenografts of HER3^+^ tumors significantly reduced tumor growth rates (Figure 8C), corresponding to a substantial reduction of ErbB2 (Figure 8D). Nonsilencing particles (H-NS) and nontargeted siRNA delivery (NT-Si) had no effect on tumor growth (Figure 8C) or ErbB2 level (Figure 8D). One tumor that did not show a change in volume after HSi-ErbB2 treatment (HSi unresponsive [UnR]; tumor volume not shown) still exhibited a considerable reduction in ErbB2 (Figure 8D) and substantial cell loss inside the tumor core compared to that in control mice receiving saline (Figure 8E). Blood chemistries showed no significant changes in liver and kidney function between HSi- and mock (saline)-treated groups (Table 3).

**Table 3.** Serum analyte panel (average ± SD) from treated mice (*N* = 5)

	Mock	HSi	H-NS
ALT (U/l)	18.25 ± 2.28	25.10 ± 3.65	23.25 ± 5.80
AST (U/l)	59.00 ± 18.34	50.33 ± 11.38	61.50 ± 39.22
BUN (mg/dl)	22.00 ± 1.41	18.33 ± 2.21	18.75 ± 1.48
CREAT (mg/dl)	0.13 ± 0.04	0.07 ± 0.05	0.10 ± 0.07

**Figure 9.**
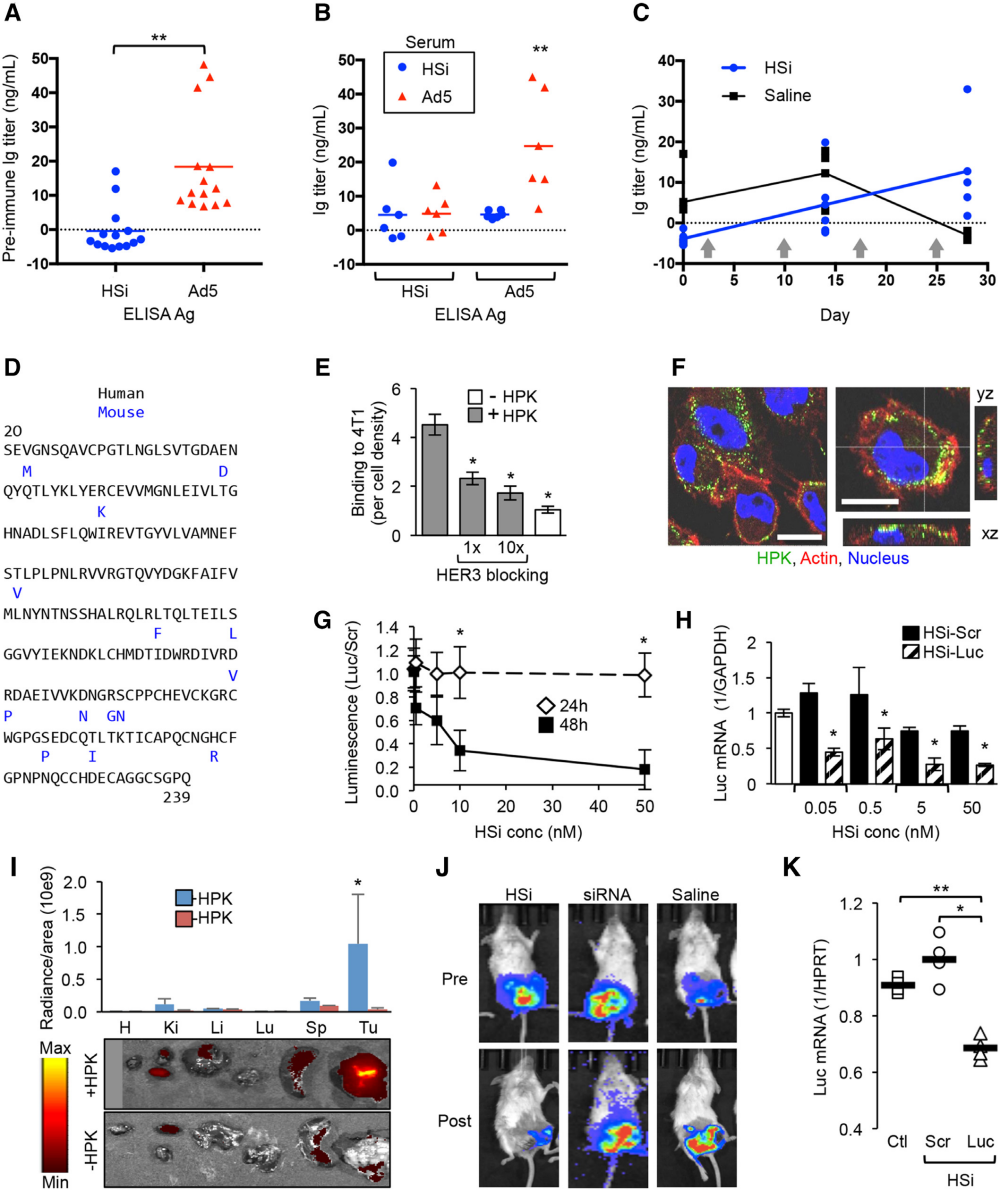
Delivery to triple-negative breast cancer cells in an immune-competent model. (A–C) ELISA of BALB/c mouse serum to detect antibodies generated against indicated antigens (HSi and Ad5) after 2× /week dosing for 4 weeks. (**A**) Ig titer measurements from preimmune serum using HSi and Ad5 as ELISA antigens. **P< 0.01 (*n* = 14). (**B**) Ig titer measurements from sera of HSi- and Ad5-inoculated mice after two inoculations, using HSi and Ad5 as ELISA antigens. **P< 0.01 versus all other combinations of serum and antigen (*n* = 6). (**C**) Ig titer measurements at sequential time points comparing sera from HSi- and mock (saline)-inoculated mice. Arrows indicate time points of inoculations (*n* = 6). (**D**) Amino acid sequence alignment of domains I–II (amino acids 20–239, heregulin-binding domain) of human and mouse HER3. Blue residues indicate amino acid differences. (**E**) Binding of HPK to 4T1 mouse triple-negative breast cancer cells. *P< 0.05 versus HPK alone (*n* = 3). (**F**) Uptake and intracellular trafficking of HPK in 4T1 mouse mammary tumor cells. Side views of cells are shown in *xz* and *yz* planes. Bars, 8 μm. (**G**) Luminescence measured from 4T1-Luc cells at indicated time points after exposure to HSi-Luc. Measurements are shown normalized to delivery of a scrambled siRNA sequence (HSi-Scr). *P< 0.05 (*n* = 3). (**H**) qPCR of luciferase mRNA isolated from 4T1-Luc cells at 48 h after treatment with HSi-Luc or HSi-Scr. *P< 0.05 versus each corresponding HSi-Scr concentration (*n* = 3). (**I**) Biodistribution of NIR-labeled oligoduplex probe alone (–HPK) or delivered by HPK (+HPK). H, heart; Ki, kidney; Li, liver; Lu, lung; Sp, spleen; Tu, tumor. *P< 0.05 versus (–HPK) and all other tissues (*n* = 3). (**J**) Luminescence imaging of representative immune-competent mice bearing orthotopic 4T1-Luc tumors before (pre) and at ∼30 h after (post) a single tail vein injection of HSi-Luc or siRNA alone (0.087 mg/kg siRNA per injection). (**K**) qPCR of mRNA isolated from 4T1-Luc tumors at 72 h after a single tail vein injection of HSi-Luc or HSi-Scr (0.087 mg/kg siRNA per injection). Ctl, control (saline injected) treatment. *P< 0.05; **P< 0.01 (*n* = 4).

To evaluate immunogenicity, we examined sera from non-tumor-bearing immunocompetent mice after exposure to HSi or control inoculants. Naïve (preimmune) mice had a significant pre-existing serum response to the control antigen, Ad5, and no significant reactivity to HSi (Figure 9A). A repeat inoculation of HSi at 10 times the therapeutic dose given to tumor-bearing mice did not produce detectable HSi-binding antibodies, whereas Ad5 treatment generated virus-binding antibodies that did not cross-react with HSi (Figure 9B). After four inoculations, HSi did not result in a significant generation of reactive antibodies in comparison to that in mock (saline)-treated mice (Figure 9C).

We also examined siRNA function in an immune-competent environment using a syngeneic model of HER3^+^ tumors. To do so, we first needed to verify whether HPK cross-reacts with mouse HER3. A sequence comparison of the ligand (i.e., heregulin)-binding regions of mouse and human HER3, specifically, extracellular domains I and II (65), showed a high level of amino acid sequence identity (94%) (Figure 9D). The 4T1 mouse mammary tumor line is a well-established model of triple-negative breast cancer (66), and we found that these tumor cells express considerable levels of HER3 on the cell surface (Supplementary Figure S2). HPK bound to these cells in a manner that was competitively inhibited by preadsorption to human HER3 peptide (Figure 9E). HPK was also taken up into 4T1 cells upon binding (Figure 9F). Together, these findings indicate that HPK cross-reacts with both mouse and human HER3. As 4T1 cells lack ErbB2 expression, we used cells stably expressing luciferase (4T1-Luc) and examined the efficacy of HSi for delivering luciferase siRNA (HSi-Luc). HSi-Luc significantly reduced luciferase luminescence (Figure 9G) and mRNA expression (Figure 9H) in cultured 4T1-Luc cells. As further *in vivo* validation, a single systemic administration of HPK delivering a labeled oligoduplex probe preferentially accumulated in orthotopic 4T1 tumors compared to nontumor tissue and in comparison to the probe alone (Figure 9I). Systemic delivery also yielded considerable reduction of tumor luminescence (Figure 9J) corresponding to a significant reduction in Luc mRNA expression (Figure 9K) in tumors from 4T1-Luc xenografts. Altogether, the results demonstrate that HSi-mediated delivery of siRNAs is effective and targeted to cells with high expression of HER3.

## DISCUSSION

The results presented here show that HPK is a HER3-targeted biocarrier for systemic homing of RNAi to HER3-expressing tumors—including triple-negative breast tumors—*in vivo*. The combination of MD simulations and functional assays revealed that HPK forms endosomolytic capsomeres that assemble with siRNA into serum-stable nucleocapsids whose ligand multivalencies hinder immune recognition while facilitating robust receptor interaction and cell uptake. We also showed that HPK cross-reacts with both human and mouse HER3 and mediates the delivery of RNAi to both human and mouse HER3-expressing tumors *in vivo* while avoiding off-target toxicity and immunogenicity. The therapeutic benefit of this application was demonstrated by the delivery of ErbB2 siRNA in a HER3-expressing tumor model, resulting in a reduced tumor burden compared to that in experimental controls.

Effective transfer of siRNA requires delivery into the cytoplasm and hence a mechanism for penetrating the plasma membrane. Several lines of evidence indicate that HPK provides this function. First, the basic foundation upon which HPK was built is the Ad5 capsid PB, which was functionally shown to bear cell-penetrating properties (33,52,58) despite an unknown mechanism of membrane destabilization. Intracellular trafficking studies of subgroup C viruses, including Ad2 and Ad5 which share high sequence identity (67), have shown that the viral particles attach to dynein motors after endosomal escape and are ferried along intact microtubules as naked particles toward the nuclear periphery (61). These particles appear as discrete puncta undergoing minus-end directed movement in fluorescence-based imaging studies (68,69). Our studies showed that recombinant soluble Ad5 PB recapitulates the trafficking of the whole virus and relies on similar microtubule-dependent transit through the cytoplasm (52). As the cytoskeleton connects organelles and mediates transport between subcellular compartments, the cytoskeletal trafficking machinery is an effective means by which viruses can deliver nucleic acid cargo to intracellular targets (70). By contrast, free nucleic acids injected into the cytoplasm exhibit little movement in the cytosolic milieu and become targeted by cytosolic nucleases (71,72), which would likely impede gene-silencing strategies targeting mRNA transcripts.

The cytoskeletal association of viral-derived proteins like the PB is thus an expected and advantageous feature after endosomal escape. Here, we observed a significant association of HPK with the cytoskeleton, similar to that for the PB and whole Ad (52,59). We also observed that after transiently overlapping with early endosomes, the majority of internalized HPK avoids late endosomes-lysosomes and accumulates intracellularly, becoming increasingly disengaged from both early and late endosomal compartments. These findings are consistent with our previous studies showing that HPK (also known as HerPBK10) (44) delivers anionic molecules—including nucleic acids and sulfonated corroles that would otherwise be repelled by the negatively charged cell membrane—to the cell cytoplasm after endocytosis into acidifying vesicles (51,54). Our previous findings show that removal of the PB domain prevents cytoplasmic entry of anionic corroles (51), suggesting that the PB domain of HPK mediates the endosomolytic process necessary for siRNA delivery. In support of this, we and others have demonstrated that recombinant soluble PB enters the cell cytoplasm after endocytic uptake (52,58). However, the mechanism by which this takes place was unknown. Our studies here suggest that the capsomere barrel formed by the PB domain of HPK acts as a pH-sensing pore whose protonation triggers disassembly and exposure of buried domains. This is evidenced by the identification of titratable histidines and other charged residues lining the barrel that considerably increase the local positive charge upon protonation at low pH. This is predicted to induce charge-mediated repellence of constituent monomers, and in agreement, we show here that lowering the pH reduces HPK oligomers to lower MW species consistent with monomers.

Our analyses also support previous crystal structure studies of the PB showing that the domains mediating pentamerization of the PB are largely hydrophobic (48). Hence, their exposure upon disassembly of the capsomere enables binding to the endosomal membrane, possibly leading to its destabilization. Indeed, both HPK and wild-type PB enter nonmembrane subcellular compartments, with some slight differences. HPK is predicted to undergo higher protonation than PBK on the basis of the analysis of titratable residues. In agreement, these proteins exhibited different sensitivities to an acidified environment, with HPK showing a response (a reduction in particle size) to a slightly milder pH environment (pH 6–7) than PBK (pH 5–6). If this transition is indeed associated with endosomolysis, these findings predict that HPK should escape from early endosomes (pH 6–6.5), whereas wild-type PB would escape from more mature endolysosomes (pH 5–5.5). This would be consistent with our previous studies using the fluorescence lifetime shift of attached corroles to determine that HPK transits through a slightly acidifying vesicle compartment before cytosolic entry (51). Overall, these findings open the door to further studies interrogating the relationship of the capsomere structure to pH-triggered subcellular dynamics and have generated two important findings. First, we have begun to uncover how the PB mediates endosomolysis. These findings have implications regarding the widely used Ad delivery vector (60,68,73), for which the efficiency of cell penetration has been attributed to the PB (58,74,75). An understanding of the mechanism by which the PB disrupts the membrane may pave the way for strategies to modulate this activity in both viral and nonviral vectors and thereby influence potential therapeutic applications. Second, our findings show evidence of HPK-cellular interactions mediated in part by the PB domain that promote the delivery of siRNA cargo to the appropriate subcellular environment.

The barrel structure formed by self-assembled HPK places the nucleic acid binding moieties at one end of the barrel, promoting the electrostatic attraction of siRNA at this same barrel façade and thus the capsomere packing around the siRNA cargo. Indeed, electron microscopy showed that HPK exposed to siRNA forms spherical particles comprised of what appear to be multimerized capsomeres. These capsomeres surround (encapsidate) the siRNA, providing protection from serum nucleases upon assembly with HPK. Whereas nucleic acids, including unmodified siRNA, are rapidly degraded in serum (76), the protection seen after assembly suggests that our payloads arrive at target tissues largely intact. Our structural modeling predicts a minimum nucleocapsid composition of 12 HPK pentamers, in agreement with the dodecahedral structures known to form from recombinant soluble Ad3 PB protein (32). However, the encapsidation of siRNA may require additional capsomeres to accommodate the cargo, thus forming spheres with diameters that may be slightly larger than that expected for (HPK)_60_. The multimerization of capsomeres should create ligand multivalency if indeed the ligands remain exposed on the nucleocapsid particle. In support, we found that the assembled particles taken up by tumor cells clustered HER3 to a greater extent than free HPK. These findings agree with the notion that capsomere assembly with the siRNA cargo results in exposure and display of the targeting ligands over the surface of the assembled particle. This, in turn, may prevent immunorecognition of the PB domain through steric hindrance. Accordingly, the HPK capsomere alone partially reduced polyclonal antibody recognition of the PB, which was further reduced by assembly into nucleocapsids. These findings suggest that the PB domain becomes immunologically masked when assembled into particles, by being buried in the nucleocapsid, being blocked by exposed ligands, or both. Immune masking of the PB via steric hindrance by other capsid proteins on the assembled Ad capsid has been described before (77,78) and could apply here via the heregulin ligands extending from the particle surface. An advantage of this is the potential to appear as a naturally occurring ligand in the body, thus providing a stealth approach to targeting. This may account for the low immunogenicity observed here, though further comparisons—to free HPK or PB, perhaps—may be warranted in future studies. Notably, our immunogenicity studies entailed inoculation with the particle at 10 times the dosage used for therapeutic efficacy, equating to a number of PB molecules (∼10^13^) roughly an order of magnitude higher than for the control inoculant, Ad5 (∼10^12^). Hence, the conditions for testing immunogenicity were appropriately rigorous.

To properly assess the ability of HPK to deliver siRNA to mouse tumors in an immunocompetent model, we first confirmed that HPK cross-reacts with mouse HER3, which was expressed at considerable levels on the surfaces of mouse 4T1 tumors (triple-negative mammary tumors). These findings correspond to the high sequence identity between the ligand binding regions of mouse and human HER3. This cross-reactivity not only enabled us to examine tumor targeting in an immune-competent environment but also raises the potential clinical relevance of our existing findings in xenograft models: HPK particles preferentially accumulated in human-derived HER3-overexpressing tumors despite the cross-reactivity with endogenous mouse HER3. This is a consideration that may be overlooked with the use of xenograft models in preclinical testing of targeted therapies that are specific to human antigens, which limits the extent to which tumor targeting can be appropriately interpreted. In many such cases, ‘tumor targeting’ may be more validly interpreted as ‘human-antigen targeting.’ Additionally, although the potential leakiness of tumor vasculature, known as enhanced permeability and retention (79,80), can contribute to tumor-preferential accumulation *in vivo*, we show here that HPK particles preferentially accumulated in the high HER3-expressing tumor rather than the low HER3-expressing tumor present on the same mouse. Taken together with the HER3 binding specificity of HPK, its blocking *in vitro* by HER3 peptide, overlap with HER3 on cell and tissue specimens, and the comparatively higher HER3 expression on tumor tissue, these studies strongly suggest that tumor targeting by HPK is directed predominantly by ligand-receptor binding to HER3-dense cells.

The therapeutic efficacy tested in this study was limited to the delivery of siRNA against ErbB2. This gene was a convenient target to prove the principle that HPK can deliver siRNA to HER3-expressing tumors and because ErbB2 amplification is frequently associated with HER3 coexpression (20). Additionally, we wanted to use a siRNA sequence known to affect the growth of such tumors (63,64). Although we were unable to use this same siRNA to test therapeutic efficacy in an immunocompetent triple-negative breast cancer model, we showed the proof of principle that HPK particles delivered RNAi for a reporter gene, luciferase, in this model. Ongoing studies are investigating potential siRNA species that could be therapeutically useful for this model and may rely on upstream regulators whose silencing would have considerable impact on tumor progression (81,82). Additional ongoing studies are investigating the influence of HPK itself on therapeutic impact. In particular, the seemingly modest but transient attenuation of tumor growth in mice by ‘empty’ HPK particles (i.e., H-NS) may be consistent with the temporary attenuation of HER3 signaling observed at early time points after receptor binding in our recent study (21), which in turn may be supported by the robust sequestration of HER3 by HPK particles observed in the present study, as well as the subsequent PB-mediated disruption of endosomes. Additional mechanistic understanding of cellular interactions of HPK and the PB may enable further design modifications to modulate potency.

The field of oligonucleotide delivery joins other fields in the ongoing concern regarding reproducibility and rigor in experimental design, execution, and analysis (83,84). Thus, we carefully conducted our experiments with rigor and transparency. Examples of this can be seen in our efforts to keep our studies unbiased by appropriately randomizing our animal subjects before treatment regimens, acquiring data in a blinded fashion, and sharing our complete methodology and all sequences to enable replication of experimental procedures. Where possible, we used alternative supplementary assays to validate our findings and ensure that interpretations were appropriate. Furthermore, to ensure reproducibility, we performed statistical power analyses *a priori* to determine appropriate sample sizes and repeated experiments independently in triplicates at the minimum. We hope these efforts undertaken at a more open and rigorous experimental design contribute to the translation of this and similar technologies into helpful novel therapeutics.

In conclusion, this study showed that the systemically administered HER3-targeted biocarrier, HPK, delivers RNAi to HER3-expressing tumors, with low to undetectable off-target toxicity and immunogenicity. We showed that HPK forms endosomolytic capsomeres that encapsidate siRNA into serum-stable particles with ligand multivalencies contributing to reduced immune recognition and robust receptor interaction, thus promoting stealth-like activity. Our demonstration that HPK recognizes both human and mouse HER3 enables testing in both xenograft and syngeneic immunocompetent tumor models. Additionally, this cross-reactivity provides a rigorous testing ground for assessing targeting to HER3-dense tissue. The overexpression of HER3 in a growing array of tumor types (1–13) indicates that the HPK-mediated targeting described here could be of broad potential benefit. The association of HER3 with resistance to growth factor inhibition (1,2,7,14–18) suggests that HPK-mediated delivery of alternative cargo such as siRNA provides a possible strategy for addressing tumors with few to no clinical options, especially since HER3 bears no inherent kinase activity that can be blocked by conventional inhibitors (19,20). Hence, the delivery of siRNA therapeutics through HER3-mediated targeting may widen the options available for treating such cancers.

## Supplementary Material

gkz900_Supplemental_Files
