# Supplementary material for: HER3-targeted protein chimera forms endosomolytic capsomeres and self-assembles into stealth nucleocapsids for systemic tumor homing of RNA interference in vivo
Source: Nucleic Acids Res. 2019 Oct 16;47(21):11020–43. doi: 10.1093/nar/gkz900 (PMC6868389; doi:10.1093/nar/gkz900)
Supplement: gkz900_Supplemental_Files [file gkz900_supplemental_files.zip › gkz900_Supplemental_File.pdf]

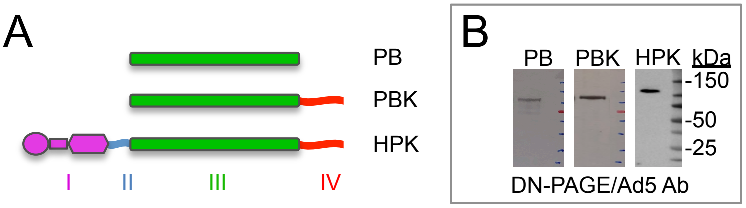

Major domains (N to C)	Domain name	Domain function
I	Her	HER3 targeting ligand
II	$(GGS)_2$	Flexible linker
III	PB	Membrane penetration
IV	K10	Nucleic acid binding

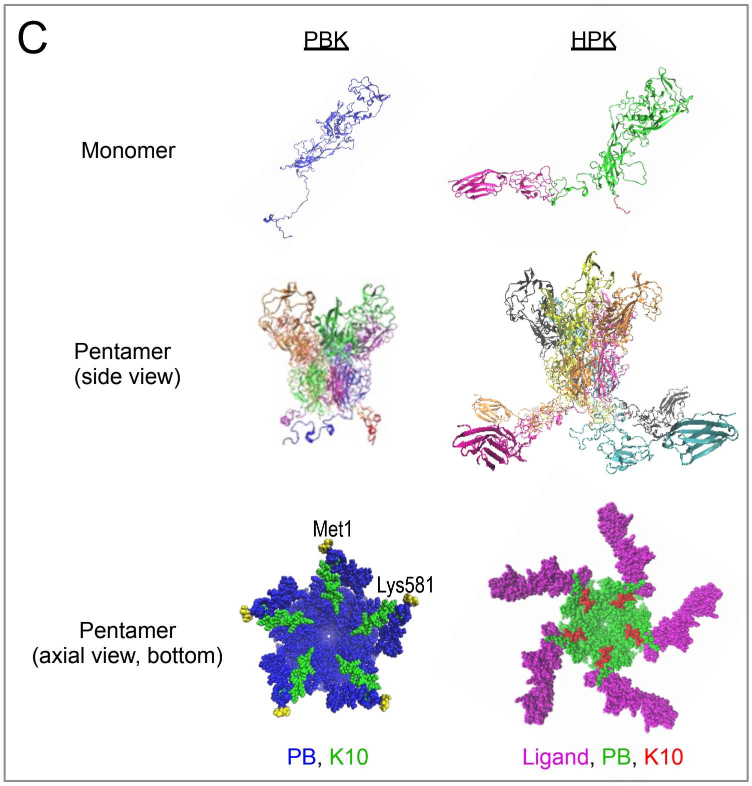

Figure S1. Summary of PB constructs, functional domains, and predicted structures. **(A)** Schematic of PB, PBK, and HPK alignments shown from amino to carboxy termini (left to right). Major domains are highlighted in different colors and assigned Roman numerals, with corresponding descriptions provided in table below schematic. (**B**) Western blots of indicated recombinant protein subject to denaturing (DN) SDS-PAGE followed by transfer to nitrocellulose membrane and immunodetection using a polyclonal antibody recognizing the PB domain (rabbit anti-Ad5; Abcam [ab6982]). (**C**) Summary of PBK and HPK structures generated by MD simulation, showing monomers, pentamers (each monomer indicated by a different color), and highlighted functional domains in the pentamers.

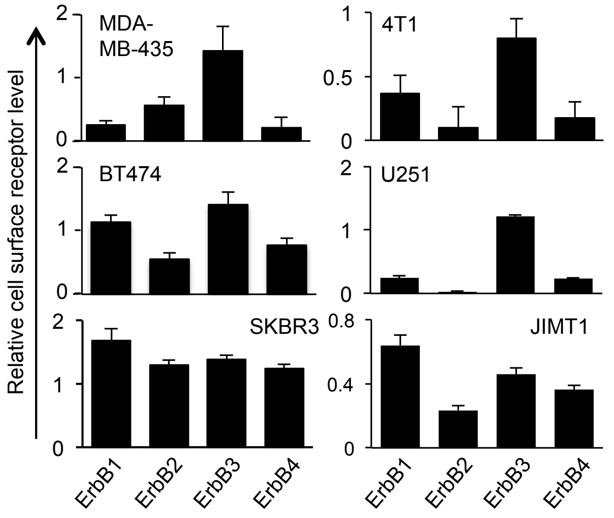

Figure S2. HER/ErbB receptor levels on different tumor lines. Relative cell surface levels of ErbB1 to ErbB4 detected by cell surface ELISA. Each receptor level signal is normalized by cell density detected by crystal violet staining.

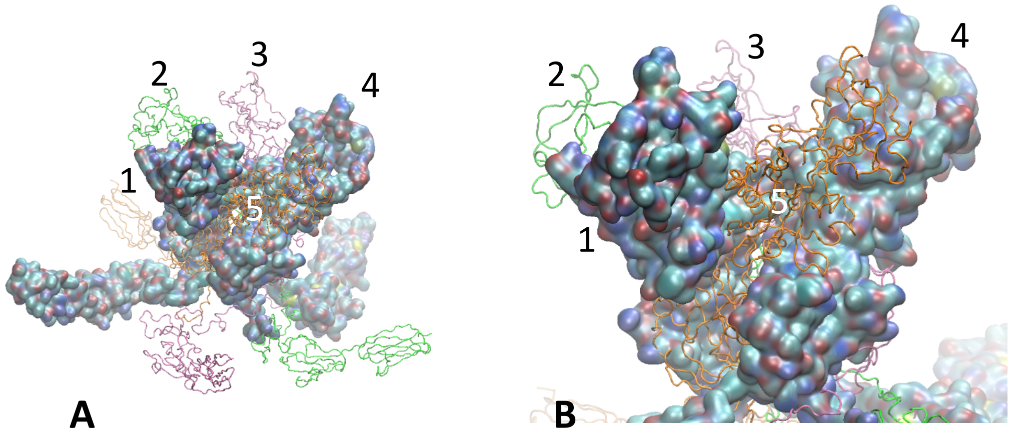

Figure S3. Buried hydrophobic surface area in the HPK particle. (**A**) Diagonal view of the HPK particle showing five protomers in the pentamer labeled 1 through 5, where 1 and 4 protomer units are shown with van der Waals surface representation to highlight green hydrophobic patches and red/blue hydrophilic patches. The other protomer units (2, 3, and 5) are shown in ribbon representation. (**B**) Side view of the same representation as in panel A, with a closeup of the hydrophobic patches on protomer units 1 and 4.

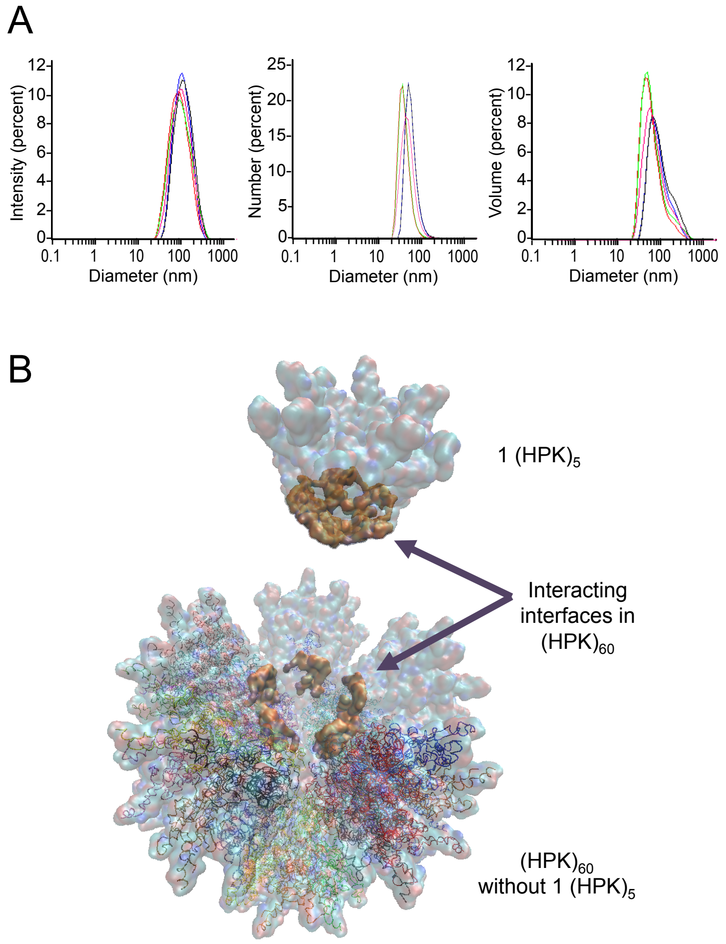

Figure S4. HPK nucleocapsid assembly. (**A**) Particle size measurements. Graphs show comparison of DLS measurements reflecting intensity, number, and mass-based parameters of the same data. Population percentage values are shown in the *y* axis. Hydrodynamic diameters are represented in a log10 scale on the *x* axis. Each analysis comprised at least three measurements per sample (represented by a different colored curve on each graph), with each measurement comprising 100 runs at an average of 34 k particle counts/s. **(B)** Structural model of $(HPK)_{60}$ highlighting the dominant interaction interface between the $(HPK)_5$ molecules. One of the $(HPK)_5$ molecules has been artificially extracted to allow visualization of the interaction interface. The full structure is shown with a molecular surface representation. The interacting residues are delineated by a gold surface and are comprised of Asp100, Arg425, and Thr427 of the HPK protein.

SUPPLEMENTARY METHODS

Hydrodynamic diameter calculation from structural modeling

This parameter was computed for chosen modeled structures by using the radius of gyration (rgyr) module of visual molecular dynamics (VMD) protein dynamics visualization and analysis program (PubMed ID: 8744570). A structure, for which hydrodynamic diameter is desired, is loaded in VMD and its radius of gyration (R_g) computed. Hydrodynamic radius (R_h) is computed from its relation to R_g using the following expression for spherical particles of uniform density:

$$R_h = \sqrt{5/3}\,R_g \approx 1.291 R_g$$

The computed R_h is then multiplied by 2 to obtain the calculated hydrodynamic diameter (D_h) for comparison with DLS measurements.

FIGURE LEGENDS TO SUPPLEMENTARY MOVIES

Movie S1. Rotating HPK pentamer in solution. Ribbon structure is shown, with each monomer delineated by a different color. (QuickTime movie converted to avi for uploading.)

Movie S2. Molecular dynamics simulation of HPK. Pentamer under physiological conditions (1 atm, 37°C). (QuickTime movie converted to avi for uploading.)

Movie S3. Rotating PBK pentamer in solution. Ribbon structure shown, with titratable amino acids represented by space-filling residues: purple, Lys/Arg; green, Asp/Glu; red, His. (QuickTime movie converted to avi for uploading.)
